# Multiscale Thermodynamics

**DOI:** 10.3390/e23020165

**Published:** 2021-01-29

**Authors:** Miroslav Grmela

**Affiliations:** École Polytechnique de Montréal, C.P.6079 suc. Centre-Ville, Montréal, QC H3C 3A7, Canada; miroslav.grmela@polymtl.ca

**Keywords:** equilibrium and nonequilibrium thermodynamics and statistical mechanics, generic, contact geometry

## Abstract

Multiscale thermodynamics is a theory of the relations among the levels of investigation of complex systems. It includes the classical equilibrium thermodynamics as a special case, but it is applicable to both static and time evolving processes in externally and internally driven macroscopic systems that are far from equilibrium and are investigated at the microscopic, mesoscopic, and macroscopic levels. In this paper we formulate multiscale thermodynamics, explain its origin, and illustrate it in mesoscopic dynamics that combines levels.

## 1. Introduction

A level of investigation is a collection of results of a certain type of experimental observations (different for different levels) made on complex systems together with a theory that allows organizing them, reproducing them, and making predictions. The theory, based on the insight inspired by experimental data and by investigating relations to nearby levels involving less or more details, offers also an understanding of the physics involved. For instance, the equilibrium level with the energy *E*, number of moles *N*, and volume *V* serving as state variables [1] and the microscopic level with the position and momenta of ∼10${}^{23}$ particles composing the macroscopic system serving as state variables are examples of two different autonomous levels of description. The latter is more microscopic (it takes into account more details) than the former. We call the latter level an upper level and the former a lower level.

Multiscale thermodynamics is a theory of the relations among different levels.

Hamilton’s mechanics, classical thermodynamics, fluid mechanics, Boltzmann’s kinetic theory, Gibbs’ equilibrium statistical mechanics, and extensive studies of the relations among them provide methods, tools, and also an inspiration to formulate a multiscale thermodynamics of which all these classical investigations are particular realizations. Multiscale thermodynamics provides a framework for investigating the static and dynamic aspects of reductions from an upper to a lower level with no constrains to the closeness to equilibrium or to the absence of external or internal driving forces.

The motivation for developing multiscale thermodynamics comes also from problems arising in nanotechnology and machine learning. For example, egg whites (as well as other polymeric fluids) behave under imposed external forces differently than water. This is because the deformations of the internal structure of egg whites (polymer macromolecules) cannot be decoupled from macroscopic deformations described by hydrodynamic fields. The fluid mechanics of complex fluids has to combine at least two different scales. Multiscale thermodynamics provides a framework for making such combinations. Its earlier versions have indeed been shown to be very useful in rheological modeling (e.g., [2] and the references cited therein). In machine learning, the objective is to extract from big data a pattern allowing making predictions. Multiscale thermodynamics was recently applied to these types of problems in [3].

Our objective in this paper is to formulate multiscale thermodynamics as a passage upper level → lower level, (in Section 2 and Section 3), to present classical investigations of mesoscopic dynamics through the eyes of multiscale thermodynamics (in Section 4), and to demonstrate its application in the mesoscopic dynamics in which the levels are combined (in Section 5).

## 2. Structures in Multiscale Thermodynamics

Let L, L, and *l* be three autonomous levels. The level L involves more details than the level L, which in turn involves more details than the level *l*. We shall call the levels involving more details upper levels or also more microscopic levels; the levels involving less details are called lower levels or also more macroscopic levels. We investigate the chain:

(1) ⟶L⟶L⟶l⟶

where ⟶ represents a reduction in which unimportant details are ignored and important overall features emerge. In the diagram (1), the way up (i.e., towards more microscopic levels) is to the left, and the way down (i.e., towards more macroscopic levels) is to the right. The level L in the diagram has two structures: one is reduced structure arising in the reduction L⟶L, and the other is the reducing structure arising in the reduction L⟶l. Every structure, both reduced and reducing, consists of a thermodynamic relation and a vector field. The former generates the geometry and the latter the time-evolution. Both depend on the other level that is involved in the reduction (i.e., at the level L in the case of the reduced structure and at the level *l* in the case of the reducing structure). Every mesoscopic level L that has neighbors on both the left and the right sides in the chain (1) has thus reduced and reducing thermodynamic relations and reduced and reducing vector fields. In general, all the reduced structures will depend on the choice of the level on its left side (i.e., the level from which it is reduced) and the reducing structures on the choice of the level on its right side (i.e., the level to which it is reducing).

The passages upper level ⟶ lower level representing the reduction process can be mathematically formulated in two ways, one called a time-evolution passage and the other a maximum entropy passage (MaxEnt passage). The former is a mathematical formulation of the time-evolution process that prepares the macroscopic systems under investigation for experimental observations at the lower level. The latter is a map transforming initial states at the level L into the final states by following the preparation process to its conclusion. In other words, the latter is a property of the solutions of the time-evolution equations introduced in the former.

If the focus of the investigation of the relations between the levels L and *l* is put on the rates of the processes involved rather than on the processes themselves, then the resulting passages and structures form what we call multiscale rate thermodynamics. The two passages, time-evolution passage and MaxEnt passage, become in rate thermodynamics the rate time-evolution passage and maximum rate-entropy passage, which we write as MaxRent passage. The structures become reducing and reduced rate structures.

Since we shall be investigating in this paper also direct links L⟶l and we shall be comparing them with the composed links L⟶L⟶l, it is more convenient to replace the chain (1) with an oriented graph in which the levels L,L,l,… are vertices and the reductions are links connecting them. The links are directed from upper to lower levels (see more in Section 4.5). Altogether, the level L in the graph is equipped with many structures depending at the levels with which it is compared (with which it is linked). The reduction represented by ⟶ has two versions: time-evolution and MaxEnt. Moreover, if the vector fields rather than state spaces are compared, then the reducing and reduced structures become reducing and reduced rate structures, and the total number of structures doubles. All the structures are not however independent. We shall see some of the dependencies below in this paper.

Before proceeding to the actual formulation of the structure and the passages, we emphasize that the term “reduction” has in this paper the same meaning as “emergence”. Some details at the upper level are lost in the reduction from an upper level to a lower level, but at the same time, an emerging overall pattern is gained. The process of reduction, as well as the processes conducive to an emergence of overall features (pattern-recognition processes) involve both a loss and a gain. The lower level is inferior to the upper level in the amount of details, but superior in the ability to display overall patterns.

### 2.1. Time-Evolution Passage

We begin by formulating the reducing structure at a level L that is being compared with a lower level *l*. Both levels L and *l* are assumed to be well established and autonomous. This means that the macroscopic systems whose behavior are found to be well described at both levels can be prepared for the level *l*. The time-evolution describing the preparation process is the reducing time-evolution taking place at L. For example, if the level *l* is the equilibrium level, the preparation process consists of leaving the macroscopic systems free of external influences and internal constraints for asufficiently long time (see more in Section 4.1).

Investigations of many pairs of levels (L, equilibrium) (see more in Section 4) revealed the following structure of the reducing time-evolution. Let *x* denote the state variable (for instance, *x* is the one particle distribution function in kinetic theory) used at the level L. The vector field generating the reducing time-evolution at the level L is the sum of two terms: one is the Hamiltonian vector field and the other the gradient vector field. The former is an inheritance of the mechanics seen at the microscopic level, and the latter drives trajectories (i.e., solutions of the governing equations) towards the time-evolution at the level *l*. Both the Hamiltonian and the gradient parts of the vector fields are gradients of a potential (i.e., co-vectors) transformed into vectors by a geometrical structure. In the Hamiltonian part, the potential is the energy and the geometrical structure the Poisson structure (in the simplest case, a skew-symmetric matrix). In the gradient part, the potential is the entropy, and the geometrical structure is the metric structure (in the simplest case, a symmetric matrix). Both geometrical structures are degenerate in order to guarantee the conservation of energy and the increase of entropy. Comments concerning the provenance of the reducing time-evolution are given in Section 4.1.

We now proceed to the mathematical formulation. The quantities characterizing states are denoted by *x* at the upper level and *y* at the lower level. All other quantities belonging to the upper level are denoted with the upper index ↑ and to the lower level with the upper index ↓. The state space at the upper level is denoted $M^{↑}$ (i.e., $x\in M^{↑}$) and the state space at the lower level $M^{↓}$ (i.e., $y\in M^{↓}$). A special notation is used for the equilibrium level; the state variables are (E,N), where *E* is the energy per unit volume and *N* the number of particles per unit volume; the state space is $M^{\left(eq\right)}$ (i.e., $\left(E,N\right)\in M^{\left(eq\right)}$). We use a shorthand notation for derivatives: $A_{x}=\frac{\partial A}{\partial x}$, where $A:M^{↑}\to R$ and $\frac{\partial }{\partial x}$ is an appropriate functional derivative in the case when $M^{↑}$ is an infinite-dimensional space.

We begin the mathematical formulation of the reducing structure at the level L with the equilibrium level playing the role of the level *l* with which we are comparing the level L. First, we need a map:

(2) $$M^{↑}\to M^{\left(eq\right)};\;\;x\mapsto \left(E^{↑}\left(x\right),N^{↑}\left(x\right)\right)$$

The time-evolution taking place in the process of preparing the macroscopic system under investigation for the equilibrium level (the reducing time-evolution) brings $x\in M^{↑}$ to $M^{↑(eq)}\subset M^{↑}$, which is in one-to-one relation with the equilibrium state space $M^{\left(eq\right)}$. Our goal now is to identify the reducing time-evolution. First, we turn to the Hamiltonian part, then to the gradient part, and finally, we combine them.

#### 2.1.1. Hamiltonian Time-Evolution

The Hamiltonian part of the time-evolution is governed by [4]:

(3) $$\dot{x}=L^{↑}E_{x}^{↑}$$

The operator $L^{↑}$ is a Poisson bivector, which means that the bracket defined by:

(4) $${\left\{A,B\right\}}^{↑}=<A_{x},L^{↑}B_{x}>$$

is a Poisson bracket (i.e., ${\left\{A,B\right\}}^{↑}=-{\left\{B,A\right\}}^{↑}$ and the Jacobi identity ${\left\{A,{\left\{B,C\right\}}^{↑}\right\}}^{↑}+{\left\{B,{\left\{C,A\right\}}^{↑}\right\}}^{↑}+{\left\{C,{\left\{A,B\right\}}^{↑}\right\}}^{↑}=0$ holds), A,B,C are sufficiently regular real valued functions of $x\in M^{↑}$, and <,> denote the pairing in the space $M^{↑}$. From the physical point of view, the bivector $L^{↑}$ expresses mathematically the kinematics of the chosen state variable $x\in M^{↑}$. For example, if x=(r,v), where *r* is the position coordinate and *v* the momentum of one particle, then $L^{↑}=\left(\begin{array}{cc} 0 & 1\\ -1 & 0 \end{array}\right)$, expressing mathematically the cotangent bundle structure of $M^{↑}$. Other examples are given in Section 4.1.

We note that the energy $E^{↑}\left(x\right)$ is conserved in the time-evolution governed by (3) since $\dot{E^{↑}}=\left\{E^{↑},E^{↑}\right\}=0$. In order to conserve other potentials in the time-evolution (3) with an arbitrary energy $E^{↑}$, the Poisson bivector $L^{↑}$ has to be degenerate. We say that $C^{↑}\left(x\right)$ is a Casimir of the Poisson bracket ${\left\{A,B\right\}}^{↑}$ if ${\left\{A,C^{↑}\right\}}^{↑}=0\;\;\;\forall A$. Consequently, $\dot{C^{↑}}=\left\{C^{↑},E^{↑}\right\}=0$. We require that the Poisson bivector $L^{↑}$ arising in the Hamiltonian part (3) of the reducing time-evolution be degenerate with the number of moles $N^{↑}\left(x\right)$ in (2), and the entropy $S^{↑}\left(x\right)$ introduced below in the gradient part of the reducing time-evolution is its Casimirs.

#### 2.1.2. Gradient Time-Evolution

The Hamiltonian dynamics (3) can be transformed into a reducing dynamics by making the following three-step reduction: (Step 1) All trajectories are found (i.e., all solutions of (3) passing through all $x\in M^{↑}$ for an ensemble of $E^{↑}\left(x\right)$ are found). The collection of all such trajectories is called a phase portrait. (Step 2) A pattern is extracted in the phase portrait. (Step 3) The pattern is interpreted as a phase portrait of the dynamics at the lower level *l*. Let us assume that the above three steps have been made. The result is expressed in the reducing time-evolution. By following it to its conclusion, we arrive at the level *l* (i.e., in the context of this section, at the equilibrium level). The equation governing the reducing time evolution is (3) modified by including a seed of dissipation. The dissipation makes unimportant details disappear (this is the loss in the reduction) and makes the pattern emerge (this is the gain in the reduction). How do we formulate the dissipation?

The most significant contribution of classical thermodynamics is the MaxEnt principle (see more in Section 4). The pattern is revealed and unimportant details discarded by maximizing a new potential $S^{↑}\left(x\right)$ called a reducing entropy. The role of the new potential $S^{↑}\left(x\right)$ in mechanics is to reveal some overall features of solutions to its governing equations. It is thus a potential that feels already some overall features of solutions and feeds them back to the initial upper vector field (see more in Section 4.1).

When reaching the lower level, the reducing entropy becomes, at the lower level, the reduced entropy $S^{↓}\left(y\right)$, where *y* is the state variable used at the lower level. In particular, if the lower level is the equilibrium level, then $S^{↓}\left(E,N\right)$ is the classical equilibrium entropy.

The simplest time-evolution making the entropy $S^{↑}\left(x\right)$ grow is the gradient dynamics [5,6]:

(5) $$\dot{x}=\Lambda^{↑}S_{x}^{↑}$$

where $\Lambda^{↑}$ is the positive definite operator. Indeed, (5) implies:

(6) $${\dot{S}}^{↑}=<S_{x}^{↑}\Lambda^{↑}S_{x}^{↑}>>0$$

where <,> denotes the pairing in $M^{↑}$.

A straightforward generalization of (5) is a dissipation-potential gradient dynamics (see more in Section 4.4):

(7) $$\dot{x}={\left[\Xi_{x^{*}}^{↑}\left(x;X\right)\right]}_{x^{*}=S_{x}^{↑}}$$

where $\Xi^{↑}$, called a dissipation potential [7], is a real-valued function of (x,X) such that:

(8) $$\begin{array}{ccc} & & \left(i\right)\;\;\Xi^{↑}\left(x,0\right)=0\\ & & \left(ii\right)\;\Xi^{↑}\;\;reaches\;\;its\;\;minimum\;\;at\;\;X=0\\ & & \left(iii\right)\;\Xi^{↑}\;\;is\;\;a\;\;convex\;\;function\;\;of\;\;X\;\;in\;\;a\;\;neighborhood\;\;of\;\;X=0\\ & & \left(iv\right)\;X\;\;is\;\;a\;\;linear\;\;function\;\;of\;\;x^{*}\;\;such\;\;that\\ & & \;\;\;\;\;\;\;\;\;\;<x^{*},\Xi_{x^{*}}>=a<X,\Xi_{X}>,\;\;where\;\;a>0 \end{array}$$

We note that the potential that generates the time-evolution (7) is the entropy S(x). The dissipation potential is a different type of potential. It does not generate the time-evolution, but it plays the role of the geometrical structure transforming the gradient $S_{x}\left(x\right)$ of the entropy (that is a co-vector) into a vector field. The right-hand side of (7) becomes the same as the right-hand side of (5) when $X=x^{*}$ and $\Xi^{↑}=\frac{1}{2}<X,\Lambda^{↑}X>$. Regarding the requirement (iv) in (8), we note that it is obviously satisfied for $X\left(x^{*}\right)=x^{*}$. In the case of *x* being a field (i.e., a function of the position coordinate *r*), then the property (iv) is for example satisfied for $X=\nabla x^{*}$ provided the boundary condition guarantees the disappearance of integrals over the boundary. An example illustrating the requirement (iv) in the case when (x= one particle distribution function) is presented in Section 4.1 in (41).

A real-valued function $C^{↑}\left(x\right)$ for which $X(C_{x}^{↑})=0$ is called gradient Casimirs. They are conserved (due to the property (iv) in (8)) in the dissipation-potential gradient time-evolution (7).

The inequality (6) becomes:

(9) $${\dot{S}}^{↑}=<S_{x}^{↑}{\left[\Xi_{x^{*}}^{↑}\left(x;x^{*}\right)\right]}_{x^{*}=S_{x}^{↑}}>=a{\left[<X^{*},\Xi_{X^{*}}>\right]}_{x^{*}=S_{x}^{↑}}>\geq 0$$

where the equality holds for the dissipation equilibrium states:

(10) $$M^{↑(deq)}=\left\{x\in M^{↑}|\Phi_{x}^{↑(diss)}=0\right\}$$

The upper thermodynamic potential $\Phi^{↑}$ is given by:

(11) $$\Phi^{↑}\left(x;C^{*}\right)=-S^{↑}\left(x\right)+<C^{*},C^{↑}\left(x\right)>$$

and $C^{*}$ can be seen as being Lagrange multipliers since (10) can be read as maximization of the entropy $S^{↑}\left(x\right)$ subject to constraints $C^{↑}\left(x\right)$.

If the thermodynamic potential $\Phi^{↑}\left(x;C^{*}\right)$ is convex, then the inequality (9) makes it possible to consider $\Phi^{↑}\left(x;C^{*}\right)$ as a Lyapunov function for the approach (as t→∞) of solutions to (7) to $M^{↑(deq)}$.

The size of the manifold $M^{↑(deq)}$ makes it also possible to give a meaning to the strength of dissipation. We say that the dissipation generated by a dissipation potential $\Xi^{\left(1\right)}$ is weaker than the dissipation generated by the dissipation potential $\Xi^{\left(2\right)}$ if $M^{(deq)1}⊃M^{(deq)2}$. The weakest dissipation is, of course, no dissipation when $M^{\left(deq\right)}\equiv M^{↑}$.

#### 2.1.3. GENERIC Time-Evolution

We now combine the seed of dissipation (7) with the Hamiltonian time-evolution (3) in a way that essential features of both mechanics (in particular, the energy conservation) and gradient dynamics (in particular, the growth of the entropy) are preserved. Both from the physical and the mathematical point of view, the combination can be best argued in the setting of the contact geometry that we present in Section 2.3 below. Here, we introduce it in the form:

(12) $$\dot{x}=L^{↑}E_{x}^{↑}+{\left[\Xi_{x^{*}}^{↑}\right]}_{x^{*}=S_{x}^{↑}}$$

which is called GENERIC (General Equation for Nonlinear Equilibrium Reversible-Irreversible Coupling) (its provenance is recalled in Section 4). Solutions to (12) are required to satisfy the following properties:

(13) $$\begin{array}{ccc} {\dot{E}}^{↑} & = & 0\\ {\dot{N}}^{↑} & = & 0\\ {\dot{S}}^{↑} & \geq & 0 \end{array}$$

The first two conservations (conservations of the energy and of the number of moles) are dictated by mechanics. In (12), we are modifying the Hamiltonian mechanics (3) by adding dissipation, but the essence of mechanics must remain intact. The modification is made in order to bring to light the overall features of solutions to (3) and not to significantly change them. The modified Hamilton Equation (12) still represents mechanics. The total energy and the total mass conservations are essential to mechanics. The last inequality in (13) (the entropy inequality) is a new feature, brought about by the modification, which is fundamental for revealing the overall features (for proving that solutions to (12) approach equilibrium states).

Before proceeding to the proof, we note that (13) are guaranteed if both the Poisson and the gradient structures are degenerate in the sense that:

(14) $$\begin{array}{ccc} & & N^{↑}\left(x\right),S^{↑}\left(x\right)\;\;\mathrm{are}\;\;\mathrm{Casimirs}\\ & & N^{↑}\left(x\right),E^{↑}\left(x\right)\;\;\mathrm{are}\;\;\mathrm{gradient}\;\;\mathrm{Casimirs} \end{array}$$

The proof of the approach to equilibrium begins with introducing an upper reducing thermodynamic potential:

(15) $$\Phi^{↑}\left(x;E^{*},N^{*}\right)=-S^{↑}\left(x\right)+E^{*}E^{↑}\left(x\right)+N^{*}N^{↑}\left(x\right)$$

where $E^{*}\in R$ and $N^{*}\in R$. If we use the notation established in the equilibrium thermodynamics, $E^{*}=\frac{1}{T}$ and $N^{*}=-\frac{\mu }{T}$, where *T* is the equilibrium temperature and μ the equilibrium chemical potential. We want to prove that solutions to (12) approach, as t→∞ equilibrium states $\hat{x}\left(E^{*},N^{*}\right)$ that are minima of (15), i.e., that are solutions to:

(16) $$\Phi_{x}^{↑}=0$$

We thus want to prove that solutions to (12) approach the manifold:

(17) $$M^{↑(eq)}=\left\{x\in M^{↑}|\Phi_{x}^{↑}=0\right\}$$

composed of the equilibrium states $\hat{x}$. We recall that the experimentally observed approach to the equilibrium states is in equilibrium thermodynamics sometimes [8] called a zero axiom of thermodynamics. A formulation of the time-evolution passage to the equilibrium level is thus a more detailed formulation of the zero axiom.

We proceed now to recall the main steps in the proof. If $N^{↑}\left(x\right),E^{↑}\left(x\right)$ is a complete set of gradient Casimirs, then the upper reducing thermodynamic potential (15) is the same as the gradient thermodynamic potential (11). Since (due to (14)) the Hamiltonian time-evolution implies ${\dot{\Phi }}^{↑}=0$, the upper reducing thermodynamic potential (15) plays the role of the Lyapunov function for the approach to dissipation equilibrium states $M^{↑(deq)}\equiv M^{↑(eq)}$ that are the same as the equilibrium states. In this case, the inequality in the third equation is sharp; the thermodynamic potential plays the role of the Lyapunov function (provided $\Phi^{↑}$ is a convex function of *x*), and indeed, the equilibrium manifold (17) is approached as t→∞.

If however the gradient part of (12) has a larger set of gradient Casimirs than $N^{↑}\left(x\right),E^{↑}\left(x\right)$ (i.e., if the dissipation is weaker), then the gradient part of (12) drives solutions to:

(18) $$M^{↑(deq)}=\left\{x\in M^{↑}|{\left[\Xi_{x^{*}}^{↑}\right]}_{x^{*}=S_{x}^{↑}\left(x\right)}=0\right\}$$

For example (see more in Section 4.1), in the Boltzmann kinetic theory, the set $M^{↑(deq)}$ is composed of local Maxwell distribution functions and $M^{↑(eq)}$ of total Maxwell distribution functions. The inequality ${\dot{\Phi }}^{↑}\leq 0$ does not suffice to prove the approach to the manifold of equilibrium states $M^{↑(eq)}$. What is needed in addition is to prove that in the course of the time-evolution, solutions to (12) never touch $M^{↑(deq)}$. Only at the final destination, the solution to (12) settles on both $M^{↑(deq)}$ and $M^{↑(eq)}$. This phenomenon started to be investigated by Grad [9]. A complete and rigorous mathematical proof for the Boltzmann equation earned Cedric Villani the Fields Medal [10]. We shall refer to the enhancement of dissipation arising in the combined gradient and Hamiltonian dynamics Grad–Villani dissipation enhancement. It is very likely an important mechanism in the onset of dissipation. Only a seed (a nucleus) of dissipation can trigger the passage from an upper level to a lower level expressed mathematically in the reducing time-evolution.

Finally, we sum up the input and the output of the reducing time-evolution to the equilibrium level. The input consists of the reducing thermodynamic relation:

(19) $$\begin{array}{ccc} N & = & N^{↑}\left(x\right)\\ E & = & E^{↑}\left(x\right)\\ S & = & S^{↑}\left(x\right) \end{array}$$

which, if inserted into (12), implies that the manifold $M^{↑(eq)}$ given in (17) is approached as t→∞, and no time-evolution takes place at the equilibrium level, i.e.,

(20) $${\left[L^{↑}E_{x}^{↑}+{\left[\Xi_{x^{*}}^{↑}\right]}_{x^{*}=S_{x}^{↑}}\right]}_{M^{↑(eq)}}\equiv 0$$

The two potentials $\left(E^{↑}\left(x\right),N^{↑}\left(x\right)\right)$ were introduced in (2) and $S^{↑}\left(x\right)$ in (5). All three arise either from a detailed experimental investigation of the preparation process for the equilibrium (by trying to express it mathematically) or from a pattern recognition process in the microscopic phase portrait (see the beginning of Section 2.1.2).

The equilibrium thermodynamic relation:

(21) $$\begin{array}{ccc} N & = & N\\ E & = & E\\ S & = & S(E,N) \end{array}$$

is the output of the reduction. It is obtained from (19) by following the time-evolution governed by (12) to its conclusion (see more in Section 2.2).

### 2.2. MaxEnt Passage

Investigations of the process of the preparation for the equilibrium level (i.e., investigations of solutions to the upper reducing time-evolution Equation (12)) in Section 2.1.3 led us to the reducing thermodynamic relation (19). We have seen that solutions to (12) approach, as t→∞, equilibrium states $\hat{x}\left(E^{*},N^{*}\right)\in M^{↑(eq)}$ that are minima of the upper thermodynamic potential (15) (i.e., $\hat{x}\left(E^{*},N^{*}\right)$ are solutions to (16)).

We now take this result of the investigations of the process of preparation for the equilibrium level as our starting point and make the passage to the equilibrium level without an explicit reference to the preparation process itself. We thus begin with the reducing thermodynamic relation (19) and with the MaxEnt principle. Our objective is to pass to the equilibrium level and arrive at the equilibrium thermodynamic relation (21) implied by (19). The passage (19) → (21) is a mapping that, as we shall see below, is a reducing Legendre transformation. The same mapping is made in Section 2.1.3, but by following the time-evolution governed by (12). The maximization of the entropy $S^{↑}\left(x\right)$ subject to constraints $E^{↑}\left(x\right),N^{↑}\left(x\right)$, postulated in this section (MaxEnt principle), is in Section 2.1.3 a consequence of the reducing time-evolution governed by (12). Furthermore, the reducing thermodynamic relation (19) arises in Section 2.1.3 from an analysis of the process of the preparation for the equilibrium level. In this section, where we do not consider the preparation process, we have to either postulate it or obtain it by using arguments based on various interpretations of the entropy (e.g., its relation to the measure of information) that were developed mainly in the context of the Gibbs equilibrium statistical mechanics (i.e., in investigations made at the microscopic level) or in the stochastic approach to thermodynamics.

The equilibrium thermodynamic relation $S^{*}\left(E^{*},N^{*}\right)$ implied by (19) is:

(22) $$S^{*}\left(E^{*},N^{*}\right)=\Phi^{↑}\left(\hat{x}\left(E^{*},N^{*}\right);E^{*},N^{*}\right)$$

where $\hat{x}\left(E^{*},N^{*}\right)$ is an equilibrium state (i.e., a solution to (16)). The equilibrium thermodynamic relation S=S(E,N) is then obtained by the Legendre transformation. This means that:

(23) $$S\left(E,N\right)=\Phi^{*}\left(\tilde{E^{*}}\left(E,N\right),\tilde{N^{*}}\left(E,N\right);E,N\right)$$

where $\Phi^{*}\left(E^{*},N^{*};E,N\right)=-S^{*}\left(E^{*},N^{*}\right)+E^{*}E+N^{*}N$ and $\left(\tilde{E^{*}}\left(E,N\right),\tilde{N^{*}}\left(E,N\right)\right)$ is a solution to $\Phi_{E^{*}}^{*}=0,\Phi_{N^{*}}^{*}=0$. Summing up, the equilibrium thermodynamic relation S=S(E,N) is obtained from the reducing thermodynamic relation (19) by two mappings:

(24) $$\left(S^{↑}\left(x\right),E^{↑}\left(x\right),N^{↑}\left(x\right)\right)\to \left(S^{*}\left(E^{*},N^{*}\right),E^{*},N^{*}\right)\to \left(S\left(E,N\right),E,N\right)$$

where the first mapping is the reducing Legendre transformation (22) and the second mapping is the Legendre transformation (23). We call (24) the maximum entropy principle (MaxEnt principle).

We now comment about the physical interpretation of the quantities $E^{*}$ and $N^{*}$. They appear at both the upper level in the thermodynamic potential $\Phi^{↑}$ (see (15)) and at the equilibrium level in the equilibrium thermodynamic potential $\Phi \left(E,N;E^{*},N^{*}\right)=-S\left(E,N\right)+E^{*}E+N^{*}N$.

At the equilibrium level, the quantities $E^{*}$ and $N^{*}$ are the conjugate variables to *E* and *N*, respectively, since the following relations hold: $E^{*}=S_{E}\left(E,N\right)$ and $N^{*}=S_{N}\left(E,N\right)$. They play a very important role in the equilibrium thermodynamics since they can be easily measured. The measurement of $E^{*}$ (and consequently, of the temperature *T* since $E^{*}=\frac{1}{T}$) is made possible by the ubiquity in nature of membranes, which either pass freely or prevent completely the passage of the internal energy *E*. If a macroscopic system is put into contact with another macroscopic system called a thermometer in such a way that the internal energy freely passes freely between the system and the thermometer and both the system and the thermometer are surrounded by the membrane that blocks the passage of the internal energy, then, due to the maximization of the entropy in the equilibrium states reached as t→∞, the temperature of the system becomes the same as the temperature of the thermometer. The temperature of the thermometer is then made visible through a known relation between the temperature and another state variable of the thermometer (e.g., volume or pressure) that can be directly observed. The existence of membranes that freely pass or block the passage of the mass then similarly makes it possible to measure $N^{*}$. Moreover, since $E^{*}=S_{E}>0$, there is a one-to-one relation between the equilibrium thermodynamics formulated in terms of (S(E,N),E,N) and (E(S,N),S,N). Using the terminology of Callen [8], the former formulation is called an entropy representation and the latter an energy representation of the equilibrium thermodynamics.

At the upper level, the quantities $\left(E^{*},N^{*}\right)$ play only the role of the Lagrange multipliers in the maximization of the reducing entropy $S^{↑}\left(x\right)$. They are no longer conjugate variables, and they cannot be simply measured at the upper level. This is the well-known problem with the definition and measurements of the temperature at mesoscopic levels (including the levels used in direct numerical simulation).

The observations that we just made about $\left(E^{*},N^{*}\right)$ are also related to the relation between the entropy representation (in which (E,N) are independent state variables) and the energy representation (in which (S,N) are independent state variables) in the classical equilibrium thermodynamics (see [8]). Due to the positivity of the absolute temperature $T={\left(S_{E}\left(E,N\right)\right)}^{-1}$, these two representations are interchangeable in the classical equilibrium thermodynamics. The equilibrium fundamental thermodynamics relation can be given either in the form (21) or in the form (N=N,E=E(S,N),S=S). This exchangeability of entropy and energy representations extends to fluid mechanics (with the fields of mass, energy, and momentum playing the role of state variables) only under the local equilibrium assumption according to which the entropy field (i.e., the local entropy) is the same function of the mass and the energy fields as in equilibrium, and thus, the field of the temperature (i.e., the local temperature) is positive (see more about this point in Section 4.3.3). In the context of a general mesoscopic level with state variables *x*, the upper reducing thermodynamic relation has only one form (19); there are no energy and entropy representations.

It is also interesting to note the difference in the inclusion of the constraints in the maximization of the reducing entropy $S^{↑}\left(x\right)$ made in the MaxEnt principle and in the maximization of the same entropy made by following the reducing time-evolution. While the former is made simply by the method of Lagrange multipliers, the latter, as we see in Section 2.1.3, is made by requiring the degeneracies of the geometrical structures involved in the vector fields and by the Grad–Villani dissipation enhancement.

Still continuing with the comparison of the MaxEnt reduction (in this section) and the reduction made in the reducing time-evolution (in Section 2.1.3, we look more closely into the role of Legendre transformations. We already noted that the MaxEnt reduction (24) is a sequence of two Legendre transformations. The first one is a reducing Legendre transformation, and the second is a regular Legendre transformation. A natural question is as to whether the reducing time-evolution is in fact also a sequence (an infinite sequence) of (infinitesimal) Legendre transformations. We answer this question in the next section.

### 2.3. Contact Geometry

Having realized that the fundamental group of thermodynamics is the group of Legendre transformations, we ask the question of what is the mathematical environment in which the Legendre transformations appear as natural transformations. The geometrical structure that is preserved in the Legendre transformations is the contact structure [4,11]. We can thus suggest that the contact geometry provides a natural mathematical environment for thermodynamics. For the classical equilibrium thermodynamics, this suggestion was made in [12,13] and for multiscale thermodynamics in [14]. In this section, we only discuss the physical aspects of the contact geometry formulation of thermodynamics. Its mathematical background can be found in [4,11].

The time-evolution governed by (12) will be a sequence of contact structure preserving (Legendre) transformations if (12) is lifted into a larger space that is equipped with a contact structure and the lifted Equation (12) will generate the time-evolution that preserves it. From the side of physics, the motivation (and guidance) for this type of reformulation of (12) comes from the following considerations. In classical (both equilibrium and nonequilibrium) thermodynamics, the conjugate state variables like the temperature and the pressure play a role of the same (if not larger) importance as the energy and volume. We can therefore suggest to adopt the conjugate state variables $x^{*}$ as independent state variables. We introduce a large space $M^{↑}$ with coordinates $\left(x,x^{*},z\right)$, $x\in M^{↑};x^{*}\in M^{↑*};z\in R$. The fact that *x* and $x^{*}$ are related in thermodynamics by $x^{*}=S_{x}^{↑}\left(x\right)$ suggests that its submanifold:

(25) $$M^{↑}=\left\{\left(x,x^{*},z\right)\in M^{↑}|x^{*}=S_{x}^{↑}\left(x\right);z=S^{↑}\left(x\right)\right\}$$

is both physically and mathematically significant. From the physical point of view, the thermodynamics takes place on $M^{↑}$. The mathematical significance of the submanifold $M^{↑}$ stems from the fact that $M^{↑}$ is equipped with the contact structure defined by the one-form:

(26) $$dz-x^{*}dx$$

and $M^{↑}$ is its Legendre submanifold. We recall that the Legendre submanifold is defined as a manifold on which the contact one-form equals zero. We note that ${\left[dz-x^{*}dx\right]}_{M^{↑}}=0$.

In order to include the MaxEnt reduction (19) to the contact geometry formulation, we have to still enlarge the space $M^{↑}$. We enlarge it into the space $\hat{M^{↑}}$ with coordinates $\left(x,x^{*},E^{*},N^{*},E,N,z\right)$ and equip it with the one-form $dz-x^{*}dx-EdE^{*}-NdN^{*}$. The Legendre manifold (25) turns in $\hat{M^{↑}}$ into another Legendre manifold:

(27) $$\hat{M^{↑}}=\left\{\left(x,x^{*},E^{*},N^{*},E,N,z\right)\in \hat{M^{↑}}|x^{*}=\Phi_{x}^{↑};E=\Phi_{E^{*}}^{↑};N=\Phi_{N^{*}}^{↑};z=\Phi^{↑}\right\}$$

The MaxEnt reduction takes place on $\hat{M^{↑}}$, and $\hat{M^{↑}}$ is again the Legendre manifold.

What remains is to lift (12) to $\hat{M^{↑}}$ in such a way that: (i) the one-form $dz-x^{*}dx-EdE^{*}-NdN^{*}$ is preserved in the time-evolution generated by the lifted (12), and (ii) the Legendre manifold $\hat{M^{↑}}$ is invariant in the time-evolution generated by the lifted (12), while the lifted Equation (12) restricted to $\hat{M^{↑}}$ is exactly the Equation (12). The time-evolution in $\hat{M^{↑}}$ satisfying these properties is called contact reducing time-evolution.

As for the first point, the canonical form of the time-evolution equations preserving a given (maximally non-integrable) one-form is well known [4,11]. The form resembles the form of the canonical Hamilton equations. In particular, the vector field is a gradient of a potential (called a reducing contact Hamiltonian $E^{↑}\left(x,x^{*},E^{*},N^{*},E,N,z\right)$ transformed into a vector by the contact geometrical structure (similar to how the Hamiltonian vector field (3) is the gradient $E_{x}^{↑}$ of the Hamiltonian $E^{↑}\left(x\right)$ transformed into a vector by the symplectic structure, i.e., by the bivector $L^{↑}$).

Regarding the second point, the contact Hamiltonian $E^{↑}\left(x,x^{*},E^{*},N^{*},E,N,z\right)$, identified in [14,15], is essentially the rate reducing thermodynamic potential (37) with $\Sigma^{↑}=\Xi^{↑}\left(x,x^{*}\right)-{\left[\Xi^{↑}\left(x,x^{*}\right)\right]}_{x^{*}=\Phi_{x}^{↑}}$, $W^{*}=E^{*}$, and $W^{↑}=<x^{*},L^{↑}E_{x}^{↑}>$.

Summing up: (i) the contact structure of the space $\hat{M^{↑}}$ remains unchanged during the contact reducing time-evolution; (ii) the contact reducing time-evolution takes place on the Legendre manifold $\hat{M^{↑}}$ given in (27); (iii) the geometrical structures appearing in (12), i.e., the symplectic structure expressed in the bivector $L^{↑}$ and the generalized gradient structure expressed in the reducing dissipation potential $\Xi^{↑}$, make their appearance in the contact reducing time-evolution in the reducing contact Hamiltonian $E^{↑}\left(x,x^{*},E^{*},N^{*},E,N,z\right)$.

The contact formulation is thus very satisfactory both from the physical and the mathematical point of view. The physical satisfaction comes from seeing the reducing thermodynamic relation (19) as a relation determining the manifold (the Legendre manifold (27)) on which the time-evolution takes place and seeing the symplectic and the gradient geometrical structures in the generating potential $E^{↑}\left(x,x^{*},E^{*},N^{*},E,N,z\right)$ (we recall that they enter GENERIC (12) in the geometry used to transform gradients of potentials into forces). The mathematical satisfaction comes mainly from the fact that the contact geometry of the space $\hat{M^{↑}}$ in which the contact time-evolution takes place remains unchanged during the time-evolution. From the mathematical point of view, we are thus as comfortable as we are with the Hamiltonian dynamics in the setting of the symplectic geometry and with the gradient dynamics in the setting of the Riemannian geometry. The GENERIC dynamics formulated in (12) involves two geometrical structures (symplectic and Riemannian); neither of them are preserved in the course of the time-evolution.

Finally, we also recall that the variational formulation that is well known for both the Hamiltonian dynamics and the gradient dynamics can be, in the setting of the contact geometry, extended to their combination, i.e., to the GENERIC dynamics [14]. The contact geometry provides also a natural setting for using the thermodynamic methods in the control theory [16,17].

### 2.4. Passage to a Lower Level with Lower Dynamics

So far, the lower level *l* with which we are comparing the upper level L is the equilibrium level that distinguishes itself among other levels mainly by the absence of the time-evolution. No time-evolution takes place at the equilibrium level (see (20). We replace now the equilibrium level with a general (but still lower than L) level *l* at which a time-evolution (called a lower time-evolution) takes place. What has to be changed in the investigation of the passage L→ *l*?

If both levels L and *l* are well established (i.e., well tested with the results of experimental observations), then there has to be a way to prepare the macroscopic systems under investigations for the level *l*, and such a preparation process has to be presentable as a time-evolution at the level L. In this respect, the replacement of the equilibrium level with the level *l* that involves the time-evolution does not bring any change. The question that remains to be answered is as to whether the preparation process is governed again by (12). We shall assume that it is (12) that governs the preparation process, but (12) with different potentials and geometrical structures. We recall that Equation (12) describing the preparation process at the equilibrium level has arisen as a common structure of this type of equations developed independently, by many researchers, in different times, and at many different levels (see Section 4). No such pool of equations is available for investigating the approach to mesoscopic levels *l* with the time-evolution. However, the basic physics that is behind (12) remains the same. We are looking essentially at the same preparation process except that we are interrupting it before its completion. The microscopic basis of the time-evolution describing the preparation process is again the particle Hamiltonian mechanics and the gradual disappearance of details in the preparation process that is expected to be mathematically manifested in the gradual decrease (or increase) of a Lyapunov-like potential. As for the question of what are the potentials and the geometrical structures appearing in (12) that represent a given macroscopic systems, we leave this at this point unanswered. We shall discuss some examples in Section 4 and Section 5.

There are however some important differences in the time-evolution representing the preparation for the equilibrium level and for the mesoscopic level *l* involving the time-evolution. First, the requirement (13) is weakened. The energy can be transferred from one level to another. Only the total energy is conserved. The energy $E^{↑}\left(x\right)$ does not have to be a gradient Casimir. The requirement (13) for the time-evolution describing L→l thus becomes $\dot{S}\geq 0$. A new requirement however emerges. In the reduction to the equilibrium level, solutions to the upper reducing time-evolution equations approach fixed points (see (20)). This means that the fixed points are eventually (as t→∞) reached and then never leave it. The fixed points are, of course, invariant manifolds. In other words, the approach to fixed points is automatically an approach to an invariant manifold. In investigations of the approach to a lower level with the lower time-evolution, trajectories in $M^{↑}$ approach $M^{↑(low)}\subset M^{↑}$, which is in one-to-one relation with the lower state space $M^{↓}$.

The requirement of the invariance of the manifold $M^{↑(low)}$ is now highly non-trivial. It is this requirement that makes the investigation of the reduction to a lower level with the time-evolution more difficult than the investigation of the reduction to the equilibrium level. The result of the investigation L→ *l*, where *l* involves the lower time-evolution, is not only the lower thermodynamic relation (i.e., the equilibrium thermodynamic relation when *l* is the equilibrium level), but also the lower time-evolution. Historically, in the first investigation of this type [18], known as the Chapman–Enskog method, the level L is the level of kinetic theory represented by the Boltzmann kinetic equation, and the level *l* is the level of hydrodynamics with the five hydrodynamic fields serving as state variables (see more in Section 4.1).

As in the investigation of the reduction to the equilibrium level (see (2)), we begin with:

(28) $$M^{↑}\to M^{↓(l)};\;\;x\mapsto y\left(x\right)$$

In the Chapman and Enskog investigation (see more in Section 4.1), y(x) are the hydrodynamic fields expressed in terms of the one particle distribution function.

The reducing time-evolution equation is (12) with the reducing thermodynamic relation:

(29) $$\begin{array}{ccc} & & y=y(x)\\ & & E^{↑}\left(x\right)\\ & & S^{↑}\left(x\right) \end{array}$$

Both the reducing energy $E^{↑}\left(x\right)$ and the reducing entropy $S^{↑}\left(x\right)$ are, in general, different from those appearing in (19). All reductions depend on both the upper level L and the lower level *l*. If the lower level changes, all quantities appearing in the reduction change. In particular, the energy $E^{↑}\left(x\right)$ appearing in (29) is only the energy involving the state variables that belong to *x*, but that do not belong to *y*. In order to avoid overburdening our notation, we do not show explicitly the dependence on the lower level *l*.

The reducing thermodynamic relation (29) is again obtained as a result of a pattern recognition process in the upper phase portrait, but the focus is put on a different pattern than in the the investigation of the passage to the equilibrium level. Instead of looking for the fixed points (17), we look for manifolds $M^{↑(low)}$ satisfying the following five properties:

(30) $$\begin{array}{ccc} & & \;\;\;\;\left(i\right)\;M^{↑(low)}\subset M^{↑}\\ & & \;\;\;\;\left(ii\right)\;M^{↑(low)}\;\;is\;\;in\;\;one-to-one\;\;relation\;\;with\;\;M^{↓}\\ & & \;\;\;\;\left(iii\right)\;M^{↑(low)}=\left\{x\in M^{↑}|\Phi_{x}^{↑}\left(x;y^{*}\right)=0\right\}\\ & & \;\;\;\;\left(iv\right)\;M^{↑(low)}\;\;is\;\;approached\;\;as\;\;t\to \infty \\ & & \;\;\;\;\left(v\right)\;M^{↑(low)}\;\;is\;\;maximally\;\;invariant \end{array}$$

where:

(31) $$\Phi^{↑}\left(x;y^{*}\right)=-S^{↑}\left(x\right)+E^{↓*}E^{↑}\left(x\right)+<y^{*},y\left(x\right)>$$

In the context of the reducing time-evolution Equation (12), we require that y(x) is both the Casimir and the gradient Casimir, $E^{↑}\left(x\right)$ is the gradient Casimir, and $S^{↑}\left(x\right)$ is the Casimir. The fifth requirement was not, of course, needed in the previous section. This new requirement plays now a very important role in determining the potentials appearing in (29). The precise meaning of maximally invariant (or alternatively “quasi-invariant”) used in (30) as well as the meaning of “appropriately projected” used in (33) below remains still a part of the pattern recognition analysis of the upper time-evolution that has to be investigated [2,19,20,21,22].

The output of the reduction L→l is the lower thermodynamics relation:

(32) $$S^{↓}=S^{↓}\left(y\right)$$

obtained from (29) in the same way as (21) is obtained from (19) (see more in Section 2.2) and the vector field (compare with (20)):

(33) $${\left[L^{↑}E_{x}^{↑}+{\left[\Xi_{x^{*}}^{↑}\right]}_{x^{*}=S_{x}^{↑}}\right]}_{M^{↑(low)}}$$

which, if appropriately projected on the tangent space $TM^{↑(low)}$ of the manifold $M^{↑(low)}$ and pushed forward on $M^{↓(l)}$ by the mapping (28), becomes the vector field generating the time-evolution at the lower level *l*. In this paper, we limit ourselves only to recalling the main idea behind the Chapman and Enskog analysis (see more in Section 4.1 and in [2,18,19,20,21,22,23]).

Before leaving this section, we make two remarks.

**Remark** **1.** *The original Chapman–Enskog investigation of the reduction of kinetic theory to hydrodynamics, as well as its continuation in [19] concentrate only on the derivation of the lower time-evolution generated by the vector field (33). The larger context of multiscale reductions has led us to the derivation of an additional result, namely to the reduced thermodynamics relation (32) that is associated with the lower time-evolution. In the reduction L→ equilibrium level, we obtained the equilibrium thermodynamic relation as the reduced thermodynamic relation, and the reduced vector field is not a vector field. In the reduction L→l that involves time-evolution, we obtain the lower thermodynamic relation (32) and the lower time-evolution generated by (33). The reduced thermodynamic relation (32) represents the thermodynamics at the equilibrium level that is inherited from the reduction L→ equilibrium level. The reduced thermodynamic relation (32) represents the thermodynamics at the level l that is inherited from the reduction L→l. As we saw in Section 2.3, the reduced thermodynamic relation (21) provides the lower state space $M^{↓(i)}$ with geometry.*

**Remark** **2.** *Externally or internally driven macroscopic systems are prevented from reaching the equilibrium level. Equilibrium thermodynamics does not exist for such systems. However, the behavior of externally or internally driven macroscopic systems can often be described at a mesoscopic level l. For example, the experimentally observed behavior of the Rayleigh–Bénard system (a horizontal layer of a fluid heated from below) can be described at the level of hydrodynamics (with Boussinesq equations governing the lower time-evolution). In other words, the level of hydrodynamics is well established for the Rayleigh–Bénard system. This then means that any other level L that involves more details and that allows expressing the physics of the Rayleigh–Bénard system (for example, the microscopic level) has to be reducible to the level of hydrodynamics. The resulting lower thermodynamic relation (32) implied by the reduction provides thus the thermodynamics replacing the equilibrium thermodynamics that does not exist. Summing up, if there exists a well-established mesoscopic level for an externally or internally driven macroscopic system (however far from equilibrium and however strong the external and internal driving forces are), then there also exists thermodynamics (expressed in the thermodynamic relation (32)) for such a system.*

### 2.5. Transitivity of Reductions

A single reduction L→l introduces two structures: reducing structure at the level L and reduced structure at the level *l*. The reducing structure consists of the reducing thermodynamic relation and the reducing time-evolution equation. The reduced structure consists of the reduced thermodynamic relation and the reduced time-evolution equation. Moreover, since for a given lower level *l*, there are, in general, many upper levels L from which it can be reduced, the every level has not one, but many reducing and reduced structures. In addition, by replacing the reduction with rate reduction, the number of the structures is multiplied by two.

Not all such structures are however independent. We shall now explore some of the relations among them. First, we turn to systems with no external and internal forces that would prevent the approach to equilibrium and to the chain:

(34) L⟶L⟶equilibrium

We expect that the reductions are transitive in the sense that the reduced equilibrium structure arising as a result of the gradual reduction L⟶L→equilibrium is the same as the reduced equilibrium structures obtained from the direct reductions L⟶equilibrium and L⟶equilibrium. This transitivity then implies the following relation between the reduced and the reducing entropies at the level L:

(35) $$H^{↓}\left(y\right)=S^{↑}\left(y\right)$$

where *H* denotes the entropy associated with the reduction L⟶L and *S* is the entropy associated with the reduction L⟶ equilibrium.

Gradual reductions (34) are more difficult to investigate than direct reductions. Nevertheless, we can, at least partially, illustrate the relation (35) with two examples. In both examples, the upper level L is the level of kinetic theory. The intermediate level L is in the first example the level of the classical fluid mechanics with the fields of mass, momentum, and internal energy as state variables. In the second example, the intermediate level L is the level of the extended fluid mechanics with *n* fields, which are velocity moments of the one particle distribution function (see more in Section 5).

In both examples, only the non-dissipative part of the time-evolution at the level L is considered. The passage L⟶L is, in both examples, the MaxEnt reduction (see Section 2.2), which does not explicitly involve the reducing time-evolution. The reduction L⟶ equilibrium is not made, in both examples, in the way described in Section 2.1.3, but in the way developed in the classical nonequilibrium thermodynamics (i.e., as an appearance of a companion local conservation law implied by a system of local conservation laws; see Section 4.3).

The first example is in fact a well-known result of the classical nonequilibrium thermodynamics. We begin with the reducing thermodynamic relation (29) at the level L in which $x=f\left(r,v\right);\;\;\;y\left(x\right)=\left(\rho \left(r\right),u\left(r\right),e\left(r\right)\right)=\left(\int dvf,\int dvvf;\int dv\frac{v^{2}}{2}\right);\;\;\;N^{↑}\left(x\right)=\int dr\int dvf;\;\;\;E^{↑}\left(x\right)=\int dr\int dv\frac{v^{2}}{2};H^{↑}\left(x\right)=-\int dr\int dvf\ln f$. The fields (ρ(r),u(r),e(r)) are hydrodynamics fields; ρ is the mass, ***u*** momentum, and *e* internal energy. The reducing thermodynamic potential (15) reaches its minimum at the local Maxwell distribution, and finally, the reduced entropy $S^{↓}\left(y\right)$ at the level L is the local equilibrium entropy given in (32) (see also Section 4.3.3). Neither the reducing, nor the reduced time-evolution is included in the investigation of the reduction L→l.

Next, we look at the time-evolution passage L⟶equilibrium. The non-dissipative part of the reducing time-evolution is the Euler hydrodynamics. Together with the reducing thermodynamic relation (19) in which N=∫drρ(r);E=∫dre(r) and S=∫drs(ρ,u,e;r), where s(ρ,u,e;r) is the local equilibrium entropy field, the Euler hydrodynamic equations imply (13) with the equality in the third equation. The entropy conservation ${\dot{S}}^{↓}=0$ arises in fact as a local conservation law $\frac{\partial s}{\partial t}=-\frac{\partial \left(su_{i}/\rho \right)}{\partial r_{i}}$ (see more in Section 4.3.3). This is a well-known result of the classical nonequilibrium thermodynamics. The relation (35) is thus in the context of the first illustration proven.

In the second example [24], we put the first example into the larger context of Grad’s hierarchy, which is a particular reformulation of Boltzmann’s kinetic equation in which the one particle distribution function is presented in the form of an infinite set of equations governing the time-evolution of its velocity moments (see more in Section 5). We realize that the hydrodynamic fields are the first five moments. In the context of Grad’s hierarchy, the first illustration is in fact a splitting of the infinite Grad hierarchy into two parts: the lower part is a closed system of equations governing the first five moments (the governing equations of Euler’s hydrodynamics), and the upper part is the remaining equations in the infinite hierarchy. The analysis of solutions to the upper part of the hierarchy is replaced (as was done also in the first illustration) by the MaxEnt passage (with the Boltzmann entropy) from the one particle distribution to its five moments. The second illustration of (35), worked out in [24], is thus the same as the first one, but with a general number *n* of Grad’s moments serving as hydrodynamic fields. Dreyer proved in [24] that all the results that we have recalled above in the first example for n=5 hold also for n>5.

The transitivity of rate reductions in the chain (34) is discussed in Section 3.

### 2.6. Criticality

The strength of the autonomy of a level is measured by the strength of fluctuations. The larger are the fluctuations, the less autonomous is the level. Large fluctuations indicate that the details that were ignored, in both experimental observations and in the mathematical formulation, cannot be ignored any longer. From the mathematical point of view, the loss of autonomy is manifested by the loss of the convexity of thermodynamic potentials. The regions in which this is happening are called critical regions.

Investigations of critical phenomena bring extra difficulties, but also extra simplifications. The first simplification is the mathematical universality of reducing thermodynamic potentials. As shown in catastrophe theory [25], real-valued smooth functions have in the vicinity of their degenerate critical points only a few nonequivalent forms (Landau polynomials). The second simplification is the inseparability of levels in the critical region allowing defining the critical region alternatively in terms of reductions.

The first simplification was noted by Landau [26]. Viewing his theory through the eyes of multiscale thermodynamics, we formulate it in the following three steps. (i) The equilibrium level is extended to an upper level with an order parameter, ξ, playing the role of an extra state variable. (ii) The upper reducing thermodynamic potential potential $\Phi (\xi ;E^{*},N^{*})$ has a universal form of Landau polynomials in the extra state variable ξ [25]. (iii) MaxEnt reduction of the extension formulated at the upper level to the equilibrium level implies a universal critical behavior at the equilibrium level.

This multiscale viewpoint of the Landau theory is formulated and illustrated by the van der Waals theory in [27,28]. The mathematical results of the universality of thermodynamic potentials in critical regions that have arisen in catastrophe theory [25] may become less surprising if we allegorically compare them with the very familiar observation that our two friends, Bill and Bob, are very different in many respects, but their behavior in critical situations is very similar. The criticality overrides the diversity.

The realization of the existence of the second simplification originally arose in the comparison of the predictions of Landau’s theory with the results of experimental observations. The agreement is found to be only qualitative. In order to explain it, attention was turned to the inseparability of levels at the critical point. It has been realized that the critical points themselves can be defined as fixed points of a group of transformations (called a renormalization group) representing a pattern recognition process. The fixed point, i.e., the critical point, is the point where no pattern can be recognized. This type of idea was originally formulated in the context of the Gibbs equilibrium statistical mechanics in [29]. In this formulation, the microscopic Hamiltonians approach in the pattern recognition process (consisting usually of a spatial coarse graining) fixed points. In the context of multiscale thermodynamics, the renormalization group approach to critical phenomena was formulated in [27,28]. In this formulation, the coefficients of the Landau polynomials approach the fixed points. The pattern recognition process is not the spatial coarse graining, but an extension of the original one component system to a two component system followed by MaxEnt reduction back to the original one component system. The two components are completely identical; they are distinguished only by a feature that does not influence at all the physical properties determining the dynamics (e.g., by a color).

## 3. Rate Thermodynamics

As we already emphasized several times, the reduction L→ *l* is either a mathematical formulation of the experimental investigation of the preparation process for the level *l* or, in the case when the time-evolution taking place at the level L is known, a pattern recognition process in the upper phase portrait. The recognized pattern is then the reduced phase portrait. In the search for the pattern, we have so far concentrated on the phase portrait in the state space $M^{↑}$. Alternatively, we can look at what is happening in the course of the preparation process in the space $X(M^{↑})$ of the vector fields on $M^{↑}$. Such a change in the focus of our attention is expected to help in recognizing overall features since the lift to higher order tangent spaces is in fact a way to see larger pieces of trajectories. Moreover, the recognized lower time-evolution will appear in $X(M^{↑})$ as a fixed point (as the lower vector field) and not as a quasi-invariant submanifold of the state space $M^{↑}$. It is easier to recognize fixed points than quasi-invariant submanifolds. For reasons that will appear later in the discussion of the relations between rate reductions and reductions (see also (8)), we shall observe the upper time-evolution in the space $X^{*}\left(M^{↑}\right)$ of co-vector fields rather than in the space $X(M^{↑})$ of vector fields. The elements of $X^{*}\left(M^{↑}\right)$ (denoted by the symbol *X*, i.e., $X\in X^{*}\left(M^{↑}\right)$) are physically interpreted as thermodynamic forces.

The change from $M^{↑}$ to $X^{*}\left(M^{↑}\right)$ is reflected in our terminology by adding the prefix “rate”. The reducing time-evolution in $X(M^{↑})$ is thus the reducing rate-time-evolution, and the thermodynamic relation is the rate thermodynamic relation. The elements of $X^{*}\left(M^{↑}\right)$ are denoted by *X* (i.e., $X\in X^{*}\left(M^{↑}\right)$) and $Y\in X^{*}\left(M^{↓}\right)$. The reducing rate-entropy is denoted by $\Sigma^{↑}\left(X\right)$, the reducing rate-energy by $W^{↑}\left(X\right)$, and the reducing rate-thermodynamic potential by $\Psi^{↑}\left(X;I\right)$. Similarly, $\Sigma^{↓}\left(Y\right)$ is the reduced rate-entropy and $W^{↓}\left(Y\right)$ the reduced rate-energy. The maximum entropy principle (MaxEnt principle) becomes the maximum rate entropy principle (MaxRent principle). We try to use the notation established in nonequilibrium thermodynamics (see also Section 4.3.1). We depart therefore from using $X^{*}$ to denote the conjugate of *X*. Instead, we denote the conjugates of *X*, having the physical interpretation of thermodynamic fluxes, by the symbol *J*, as is customary in classical nonequilibrium thermodynamics (see also Section 4.3.1). Similarly, at the lower level *l*, the thermodynamic forces are denoted by the symbol *Y* and its conjugates, having the physical interpretation of lower level thermodynamic fluxes, by the symbol *I*.

From the physical point of view, the preparation process for the level *l* is the same as in Section 2.4. We just observe it differently. The mathematical formulation of the MaxRent passage L→l begins with the reducing rate thermodynamic relation:

(36) $$\begin{array}{ccc} & & Y=Y(X)\\ & & W^{↑}\left(X\right)\\ & & \Sigma^{↑}\left(X\right) \end{array}$$

where $W^{↑}\left(X\right)$ is the rate of energy. The rate thermodynamic corresponding to it potential reads:

(37) $$\Psi^{↑}\left(X;W^{↓*},I\right)=-\Sigma^{↑}\left(X\right)+W^{↑}\left(X\right)W^{↓*}+<Y\left(X\right),I>$$

where $\left(W^{↓*},I\right)$ are Lagrange multipliers.

Next, we pass by the MaxRent reduction from $\Sigma^{↑}\left(X\right)$ to $\Sigma^{↓*}\left(W^{↓*},I\right)$ and finally (by the ordinary Legendre transformation) to $\Sigma^{↓}\left(W^{↓},Y\right)$. The Legendre transformations involved in the MaxRent reduction are the same as the Legendre transformations made in the MaxEnt reduction in Section 2.2.

If we compare the rate thermodynamic potential (37) with the thermodynamic potential (15), we note that the coefficient $W^{↓*}$ is in rate reductions analogous to the coefficient $E^{*}=\frac{1}{T}$ introduced in reductions. We therefore physically interpret $W^{↓*}$ as an inverse rate temperature T, i.e., $W^{↓*}=\frac{1}{T}$. In terms of the lower entropy $\Sigma^{↓}\left(W^{↓},Y\right)$, the rate temperature T becomes:

(38) $$\Sigma_{W^{↓}}^{↓}\left(W^{↓},Y\right)=\frac{1}{T}$$

The rate temperature T can be measured with a rate thermometer similarly as the temperature *T* is measured with a standard thermometer. The difference between the standard and the rate thermometers is in the walls separating the thermometers from the system whose temperature is measured. In the standard thermometers, it is a wall that freely passes or stops passing the internal energy. Such walls are ubiquitous in nature. In the rate thermometers, the walls have to freely pass or stop passing the rate of the internal energy. Such walls are certainly not ubiquitous in nature. The rate temperature remains thus still only a theoretical concept.

As for the rate time-evolution passage L→ *l*, we already noted that even if the lower level *l* involves the lower time-evolution in the space of vector fields, $X^{*}\left(M^{↑}\right)$ still approaches a fixed point (the lower vector field) and not a quasi-invariant manifold. We shall make some additional observations addressing this aspect of the reducing rate time-evolution in Section 5. Regarding other aspects of the time-evolution governing the passage $X^{*}\left(M^{↑}\right)\to X^{*}\left(M^{↓}\right)$, we conjecture that it possesses the GENERIC structure discussed in Section 2.1.3. Contrary to the passage $M^{↑}\to M^{\left(eq\right)}$ for which we have many specific examples (which have been developed independently and at many different levels) that all possess GENERIC, the argument supporting this conjecture is only the consistency that GENERIC provides for combining the time reversible and non-dissipative mechanics with a time irreversible and dissipative mechanism in which unimportant details are disappearing.

Our discussion of the rate thermodynamics remains so far in the space $X^{*}\left(M^{↑}\right)$ in which we did not relate its elements *X* to $M^{↑}$. We have so far no connection between the time-evolution of $X\in X^{*}\left(M^{↑}\right)$ and the time-evolution of $x\in M^{↑}$. These two time-evolutions become related if $X\in X^{*}\left(M^{↑}\right)$ becomes related to $x\in M^{↑}$. We have to say something about the function $X(x^{*})$. We recall that we have already addressed this function in (8), where we collected the properties of the dissipation potential. In particular, the dissipation potential $\Xi^{↑}$ has been found to depend on $x^{*}$ through its dependence on the thermodynamic force *X* and in a way that all four properties in (8) are satisfied.

In order to discuss the compatibility of the rate thermodynamics with the thermodynamics presented in Section 2, we consider a macroscopic system at three levels (34) in the absence of external and internal forces that prevent the approach to equilibrium. The difference between (34) in Section 2.5 and this section is that ⟶ in Section 2.5 represents reductions, and in this section, it represents rate reductions. The compatibility of the rate passage L→L with the passage L→ equilibrium requires:

(39) $$\Sigma^{↓}\left(Y\right)=\Xi^{↑}\left(Y\right)$$

where *Y* is the co-vector field at the level L. This compatibility relation implies then the relation:

(40) $$S^{↑}=<S_{y}^{↑}\left(y\right),\dot{y}>={\left[<y^{*},\Xi_{y^{*}}^{↑}>\right]}_{y^{*}=S_{y}^{↑}\left(y\right)}=a{\left[<Y\left(y^{*}\right),\Sigma_{Y(y^{*})}^{↓}>\right]}_{y^{*}=S_{y}^{↑}\left(y\right)}$$

between the reduced rate entropy and the entropy production, both at the level L.

The results of the classical nonequilibrium thermodynamics that gave birth to the rate thermodynamics are recalled in Section 4.3.3.

## 4. Particular Realizations of the GENERIC Structure

The unified formulation of equilibrium thermodynamics, non-equilibrium thermodynamics, equilibrium statistical mechanics, and non-equilibrium statistical mechanics provided by multiscale thermodynamics has emerged as a collection of common features extracted from a large body of investigations of macroscopic systems at many different levels ranging from the equilibrium to the microscopic. We now recall some of the principal results on which multiscale thermodynamics stands. The feedback of the abstract formulation to the investigation of some specific problems arising in hierarchy reformulations of dynamics is explored in Section 5.

The first step towards a unified viewpoint of microscopic and mesoscopic dynamics was made by Alfred Clebsch [30], who cast the Euler hydrodynamics (i.e., a continuum version of Newton’s mechanics) into the Hamiltonian form. In particular, in Arnold’s [31] formulation, the Hamiltonian fluid mechanics inspired efforts to see also other mesoscopic non-dissipative dynamical theories (including for instance kinetic theories) as particular realizations of an abstract Hamilton dynamics. A modification needed to include dissipative mesoscopic dynamics was made in [32] and later in [33,34,35]. The importance of such a unified formulation was gradually realized in [36,37]. Its usefulness, for instance in the fluid mechanics of complex fluids, was first demonstrated in [38,39]. An important step in the further theoretical development and in applications was made in [40,41] (where the acronym GENERIC (General Equation for Nonlinear Equilibrium Reversible-Irreversible Coupling) appeared for the first time) and also in [36,42]. The contact geometry formulation of GENERIC was introduced in [14]. A recent systematic presentation of multiscale thermodynamics, together with many applications, can be found in [43].

### 4.1. Boltzmann Kinetic Equation: Time-Evolution Passage

Historically, the first investigation of the time-evolution passage L→ equilibrium was made by Boltzmann [44]. The physical system in his analysis is the ideal gas, and the upper level L is the level of kinetic theory in which one particle distribution function $f\left(r,v\right)\in M^{↑}$ plays the role of the state variable (***r*** is the position vector and ***v*** the momentum of one gas particle). The power and the enormous importance of Boltzmann’s results, as well as the results obtained by his numerous followers, is not the narrow focus on the ideal gas, but the physical insight and the mathematical structure involved in the investigation.

We introduced in Section 2.1.2 the notion of the entropy $S^{↑}\left(x\right)$ as a quantity that plays in mechanics the role of revealing the overall features of the phase portrait. Following Boltzmann’s insight, the entropy arises in the investigation of gas dynamics as follows. The events in the gas time-evolution that play the most important role in determining the overall appearance of the phase portrait are binary collisions. We therefore consider the free flow of the gas particles and their binary collisions separately. The former induces directly the time-evolution of the one particle distribution function: $f\left(r,v,t\right)=f_{0}\left(\Gamma_{-t}\left(r,v\right)\right)$, where $f_{0}\left(r,v\right)$ is the distribution function at t=0 and $\Gamma_{t}\left(r,v\right)$ is the trajectory generated by $\dot{r}=\frac{v}{m};\dot{v}=0$. The latter enters the vector field on $M^{↑}$ indirectly. Complete trajectories of colliding particles are found first and then transformed into a gain loss balance-type vector field on $M^{↑}$. The transformation is, allegorically speaking, a “retouch” of the trajectories of colliding particles in which the details are ignored and only the energy and the momentum conservations are kept. The Boltzmann entropy is then born in an analysis of the solutions of the Boltzmann equation in which the time-evolution is generated by a vector field that is the sum of the Hamiltonian free flow part and the modified collision part.

In mathematical terms, the Boltzmann kinetic equation takes the form of (12) with:

(41) $$\begin{array}{ccc} & & E^{↑}\left(f\right)=\int dr\int dv\;f\frac{v^{2}}{2m};\;\;\;N^{↑}\left(f\right)=\int dr\int dvf\\ & & \left\{A,B\right\}=\int dr\int dvf\left(\frac{\partial A_{f}}{\partial r_{i}}\frac{\partial B_{f}}{\partial v_{i}}-\frac{\partial B_{f}}{\partial r_{i}}\frac{\partial A_{f}}{\partial v_{i}}\right)\\ & & \Xi^{↑}\left(f,f^{*}\right)=\int d1\int d2\int d1^{\prime }\int d2^{\prime }W\left(f;1,2,1^{\prime },2^{\prime }\right)\left(e^{X}+e^{-X}-2\right)\\ & & X=f^{*}\left(1\right)+f^{*}\left(2\right)-f^{*}\left(1^{\prime }\right)-f^{*}\left(2^{\prime }\right) \end{array}$$

where *m* is the mass of one particle. We use hereafter the summation convention over the repeated indices and the shorthand notation $1=\left(r_{1},v_{1}\right);2=\left(r_{2},v_{2}\right),1^{\prime }=\left(r_{1}^{\prime },v_{1}^{\prime }\right);2^{\prime }=\left(r_{2}^{\prime },v_{2}^{\prime }\right)$. Two particles enter the collision with coordinates 1 and 2 and leave it with coordinates $1^{\prime }$ and $2^{\prime }$. It is assumed that the particles are point particles, and their position coordinates remain unchanged in the collisions (i.e., $r_{1}=r_{1}^{\prime }=r_{2}=r_{2}^{\prime })$. The mechanics of binary collisions is introduced into the formulation of the kinetic Equation (12) with (41) in two places, First, in the dissipation potential $\Xi^{↑}\left(f,f^{*}\right)$ in the following restrictions on the choice of *W*: (i) W≠0 only if the energy and momentum are conserved, i.e., if $v_{1}^{2}+v_{2}^{2}={\left(v_{1}^{\prime }\right)}^{2}+{\left(v_{2}^{\prime }\right)}^{2}$ and $v_{1}+v_{2}=v_{1}^{\prime }+v_{2}^{\prime }$, (ii) W>0, and (iii) *W* is symmetric with respect to 1⇆2 and $\left(1,2\right)⇆\left(1^{\prime },2^{\prime }\right)$. The second place where the mechanics of binary collision enters is in the specification of the entropy $S^{↑}\left(f\right)$ that enters the dissipation potential $\Xi^{↑}\left(f,f^{*}\right)$ in the relation between *f* and $f^{*}$ (i.e., $f^{*}=S_{f}^{↑}$). The Boltzmann entropy $S^{↑}\left(f\right)=-\int dr\int dvf\left(r,v\right)\ln f\left(r,v\right)$ emerges when the form of the collision gain loss balance calculated from the collision mechanics (see, e.g., [18]) is cast into the form $\Xi_{f^{*}}^{↑}\left(f,f^{*}\right)$ (the second term on the right-hand side of (12) with $\Xi^{↑}$ given in (41)).

The form of the dissipation potential $\Xi^{↑}\left(f,f^{*}\right)$ of the collision part of the Boltzmann kinetic equation arises naturally if we regard binary collisions as chemical reactions [45,46] in which two species labeled by $v_{1}$ and $v_{2}$ react and produce two species labeled by $v_{1}^{\prime }$ and $v_{2}^{\prime }$ and vice versa. The thermodynamic force *X* is called in chemical kinetics a chemical affinity. The dissipation potential $\Xi^{↑}\left(f,f^{*}\right)$ appearing in (41) is indeed the dissipation potential arising in chemical kinetics [46] (see more in Section 4.4. The property (iv) in (8) is a straightforward consequence of the symmetries of $W(f;1,2,1^{\prime },2^{\prime })$. The coefficient *a* appearing in the property (iv) is in this example a=1/4.

We now recall some important properties of solutions to the Boltzmann kinetic equation. We begin with the global existence of its solutions that was proven in [47]. DiPerna and Lions received for this work the Fields Medal. Another Fields Medal was received by Cedric Villani [10] for proving the approach of the solutions of the Boltzmann kinetic equation to the equilibrium states.

The dissipation equilibrium manifold $M^{↑(deq)}$ (see Section 2.1.3) is composed of solutions to $\Xi_{f^{*}}^{↑}\left(f,f^{*}\right)=0$. i.e., solutions to $X(f^{*})=0$. With the Boltzmann entropy, the solutions are the local Maxwell distribution functions, which are also solutions to:

(42) $$\begin{array}{ccc} & & \Phi_{f(r,v)}^{↑(loc)}=0;\\ & & \Phi^{↑(loc)}\left(f;e^{*}\left(r\right),u^{*}\left(r\right),n^{*}\left(r\right)\right)=-S^{↑}\left(f\right)\\ & & +\int dre^{*}\left(r\right)e\left(f;r\right)+\int dru_{i}^{*}\left(r\right)u_{i}\left(f;r\right)+\int drn^{*}\left(r\right)n\left(f;r\right);\\ & & e\left(f;r\right)=\int dvf\frac{v^{2}}{2m};\;\;\;\;u\left(f;r\right)=\int dvfv;\;\;\;\;n\left(f;r\right)=\int dvf \end{array}$$

The equilibrium manifold $M^{↑(eq)}$ is composed of solutions to the Boltzmann kinetic equation reached as t→∞. These distribution functions are Maxwell distribution functions that are solutions to:

(43) $$\begin{array}{ccc} & & \Phi_{f(r,v)}^{↑}=0\\ & & \Phi^{↑}\left(f;E^{*}.N^{*}\right)=-S^{↑}\left(f\right)+E^{*}E^{↑}\left(f\right)+N^{*}N^{↑}\left(f\right) \end{array}$$

The two manifolds $M^{↑(eq)}$ and $M^{↑(deq)}$ are related by $M^{↑(eq)}\subset M^{↑(deq)}\subset M^{↑}$.

The fact that the Boltzmann kinetic equation is a particular realization (41) of the abstract GENERIC Equation (12) implies that its solutions approach the local Maxwell distribution functions (42). To prove that they approach a smaller manifold, namely the manifold composed by the Maxwell distribution functions expressing equilibrium states (i.e., solutions to (43)), requires extra effort [10]. The Grad–Villani dissipation enhancement (see Section 2.1.3), needed to narrow down the asymptotically reached manifold, arises due to the presence of the free flow in the vector field.

Beside the opportunity to investigate rigorously the approach to the equilibrium level, Boltzmann’s kinetic theory provides also an opportunity to investigate the approach to a lower level involving the time-evolution (i.e., the situation discussed in Section 2.4). The mapping (28) is chosen as follows:

(44) $$f\left(r,v\right)\mapsto \left(\rho \left(r\right),u\left(r\right),e\left(r\right)\right)=\left(\int dvmf\left(r,v\right),\int dvvf\left(r,v\right),\int dv\frac{v^{2}}{2m}f\left(r,v\right)\right)$$

The *l*-manifold $M^{↑(l)}\subset M^{↑}$ is searched by a perturbation method in which the dissipation equilibrium manifold (42) serves as its initial approximation [18]. In this initial approximation, the Boltzmann kinetic equation turns into the Euler hydrodynamic equations (i.e., into the Hamiltonian part of the hydrodynamic equations).

The Chapman–Enskog method thus begins with the dissipation equilibrium manifold (42), the Euler vector field on its tangent space, the Boltzmann entropy, and the local equilibrium reduced thermodynamic relation in the hydrodynamics state space that is implied (see (42)) by the Boltzmann entropy. The next step in the Chapman–Enskog method is a deformation of the dissipation equilibrium manifold (42), (that we now denote $M^{↑(deq0)}$) into $M^{↑(deq1)}$, which is required to be more invariant than $M^{↑(deq0)}$). We say that a manifold M⊂M is more invariant, with respect to F∈X(M), than a submanifold N⊂M if, roughly speaking, the vector field ${\left[F\right]}_{M}$ is sticking out of TM more than the vector field ${\left[F\right]}_{N}$ is sticking out of TN. The results of the investigation will still, of course, depend on the precise meaning we give to “sticking out more” and “sticking out less” (see more in [19]).

After making the first step in the Chapman–Enskog method, we obtain an appropriately deformed manifold $M^{↑(deq1)}$ with the Navier–Stokes–Fourier vector field on its tangent space and a new entropy $S^{\left(1\right)}$ (whose maximization provides $M^{↑(deq1)}$) and the new reduced thermodynamic relation corresponding to it.

The Navier–Stokes–Fourier vector field is the vector field

${\left[Boltzmann\;\;vector\;\;field\right]}_{M^{↑(deq1)}}$ that is appropriately projected on the tangent space of the manifold $M^{↑(deq1)}$ (see more details in [2,19,20,21,22]). The reducing entropy $S^{↑}\left(x\right)$ is obtained as follows. Let $M^{↑(l0)}$ be the initial manifold with which the Chapman–Enskog iterations begin. In the context of the reduction of kinetic theory to hydrodynamics, the manifold $M^{↑(l0)}$ is the manifold formed by local Maxwell distributions. The manifold corresponding to the first Chapman–Enskog approximation is denoted by $M^{↑(l1)}$. Let $S^{↑(0)}\left(x\right)$, $S^{↑(1)}\left(x\right)$ be the entropies corresponding to $M^{↑(l0)}$, $M^{↑(l1)}$ in the sense that $M^{↑(l0)}$ is formed by solutions to (16) with $S^{↑(0)}\left(x\right)$ and $M^{↑(l1)}$ is formed by solutions to (16) with $S^{↑(1)}\left(x\right)$. In the context of the reduction of kinetic theory to hydrodynamics, $S^{↑(0)}\left(x\right)$ is the Boltzmann entropy. This type of the Chapman–Enskog sequence of reducing entropies that is induced by the sequence of the Chapman–Enskog reduced vector fields was discussed in [2,20,21,22].

An alternative investigation of the reduction kinetic theory level → hydrodynamics level that begins with the Grad hierarchy formulation of the kinetic equation [48] will be discussed in Section 4.3.2 and Section 5.

Both the Chapman–Enskog and the Grad types of reductions require a complex investigation of the solutions of the kinetic equations. If we however concentrate our attention only on kinematics, then the reduction from the kinematics of the one particle distribution function expressed in the Poisson bracket (41) to the kinematics of the hydrodynamic fields expressed mathematically in the Poisson bracket (57) is completely straightforward and completely rigorous. The derivation proceeds as follows. First, we limit the Poisson bracket in (41) to functions A,B that depend on *f* only through their dependence on (∫dvf,∫dvη(f),∫dvvf), where ∫dr∫dvη(f) is a Casimir of the Poisson bracket (41). This means that we replace $A_{f}$ with $A_{\rho }+\eta_{f}A_{s}+vA_{v}$, and similarly, $B_{f}$ with $B_{\rho }+\eta_{f}B_{s}+vB_{v}$. Straightforward calculations (see [43] and the references cited therein and [49]) lead then from (41) to (57).

### 4.2. Gibbs MaxEnt Passage: Gibbs Equilibrium Statistical Mechanics

The MaxEnt passage L→l, discussed in Section 2.2, was made first by Gibbs [50] for L being the microscopic level and *l* the equilibrium level. The reducing time-evolution equation describing the preparation process for such a passage is not a part of the Gibbs analysis. The preparation process is represented only in a few requirements: the gradient part of the reducing time-evolution by a reducing entropy that is required to be maximized, the Hamiltonian part by constraints in the maximization. The applicability of the Gibbs reduction is universal.

In mathematical terms, the upper state variable is the *n* particle distribution function $f\left(1,\ldots ,n\right)\in M^{↑}$; $n∼10^{23}$ is the number of particles. The Gibbs MaxEnt reduction starts with the upper reducing thermodynamic relation:

(45) $$\begin{array}{ccc} N^{↑}\left(f\right) & = & \int d1,\ldots ,\int dnf(1,\ldots ,n)\\ E^{↑}\left(f\right) & = & \int d1,\ldots ,\int dnf(1,\ldots ,n)e(1,\ldots ,n)\\ S^{↑}\left(f\right) & = & -k_{B}\int d1,\ldots ,\int dnf\left(1,\ldots ,n\right)\ln f\left(1,\ldots ,n\right) \end{array}$$

where $k_{B}$ is the Boltzmann constant, e(1,…,n) is the energy (Hamiltonian) of *n* particles, and $k_{B}$ is the Boltzmann constant. The passage to the equilibrium thermodynamic relation (21) is made in the way described in Section 2.2.

We now compare the Gibbs MaxEnt passage to the equilibrium level with the Boltzmann’s time-evolution passage (see Section 4.1) also to the equilibrium level. Boltzmann begins with an insight into the appearance of the phase portrait of the reducing time-evolution equation. The crucial role in the emergence of the equilibrium pattern in the phase portrait is expected to be played by collisions. The part of the vector field generating the collision trajectories is thus first “pre-processed” before putting it back into the total vector field. The pre-processing consists of ignoring the details and keeping only the momentum and energy conservations. From this viewpoint, the pre-processed collision vector field takes the form of a gain-loss balance known from chemical kinetics. An investigation of the time-evolution governed by the Boltzmann equation, i.e., by the free flow vector field + the pre-processed collision vector field, reveals that the approach to the equilibrium level is driven by Boltzmann’s H-function, which we call the Boltzmann entropy. The equilibrium level can be reached by following the time-evolution governed by the Boltzmann equation or alternatively and equivalently by the MaxEnt reduction process in which the Boltzmann H-function is maximized subject to the energy and the number of moles constraints.

Gibbs also begins with an insight into the appearance of the phase portrait. However, instead of expressing it in a modification of the vector field that generates it, as Boltzmann does, Gibbs expresses it directly in the entropy that generates it in the MaxEnt reduction. Both the Gibbs entropy (that is universal at the microscopic level) and its maximization (the MaxEnt principle) are postulated. The microscopic Hamiltonian vector field is represented in the Gibbs MaxEnt reduction only in the constraint of the Gibbs entropy maximization. The energy is required to remain unchanged in the reduction. The Gibbs equilibrium pattern is also often called “ergodic” with only very vague reference to the rigorous mathematical definition of ergodicity in the theory of dynamical systems on measurable spaces [51]. The phase portrait of the ergodic (in the rigorous mathematical sense) time-evolution does possess the Gibbs pattern, but the Gibbs MaxEnt reduction applies to a much larger class of time-evolutions.

There is, of course, an enormous difference between the Boltzmann and the Gibbs approaches to the passage L→l in the domain of applicability. While the Gibbs theory is applicable to all macroscopic systems, the Boltzmann theory is applicable only to ideal gases. The pattern that in the upper-level phase space in the Gibbs theory characterizes the equilibrium level (as well as the entropy generating it in the MaxEnt reduction) is universal, but it is postulated. In Boltzmann’s theory, the pattern in the upper-level phase portrait characterizing the equilibrium level is generated by the time-evolution governed by the Boltzmann equation, but the analysis is made only for ideal gases. Nevertheless, as we have already pointed out in the previous section, the mathematical structure of the Boltzmann equation has inspired and continues to inspire investigations of the time-evolution of macroscopic systems at all levels.

#### Gibbs Time-Evolution Passage

An obvious question is: What is the Gibbs time-evolution passage that becomes the Gibbs MaxEnt passage if only an initial state and the final state reached as t→∞ are considered? This question was already asked in [52]. We continue to discuss it here. The kinematics of the N particle distribution function is expressed mathematically in the Poisson bracket:

(46) $$\left\{A,B\right\}=\int d1\ldots \int dnf\left[\frac{\partial A_{f}}{\partial r_{\alpha i}}\frac{\partial B_{f}}{\partial v_{\alpha i}}-\frac{\partial B_{f}}{\partial r_{\alpha i}}\frac{\partial A_{f}}{\partial v_{\alpha i}}\right]$$

Its derivation follows completely the derivation of the Poisson bracket for the one particle distribution function appearing in (41). The time-evolution Equation (3) corresponding to the bracket (46) is the Liouville equation [53,54,55]:

(47) $$\frac{\partial f}{\partial t}=-\frac{\partial }{\partial r_{\alpha i}}\left(f\frac{\partial E_{f}}{\partial v_{\alpha i}}\right)+\frac{\partial }{\partial v_{\alpha i}}\left(f\frac{\partial E_{f}}{\partial r_{\alpha i}}\right)$$

We note that the Liouville Equation (47) is a linear equation independent of the complexity of the interaction among the particles. The Liouville lift transforms the very nonlinear particle dynamics in the finite-dimensional space with (1,…,n) as its elements into a linear dynamics in the infinite-dimensional space with (f(1,…,n) as its elements.

Next, we follow Boltzmann and introduce dissipation. From the physical point of view, we need to identify an event (or events) in which unimportant details are generated. Such events are analogical to binary collisions in ideal gases. Let such an event be identified. The vector field generating it is replaced by an in-and-out balance generated by mappings:

(48) $$\Upsilon \left(1,\ldots ,n\right)=\left(1^{\prime },\ldots ,n^{\prime }\right)$$

that we call Boltzmann regularization mappings. In Boltzmann’s analysis of an ideal gas, the mapping Υ represents transformations of incoming momenta $\left(v_{1},v_{2}\right)$ of a binary collision into the outgoing momenta $\left(v_{1}^{\prime },v_{2}^{\prime }\right)$. The invariants of Υ are $(r_{1},\left(r_{1}-r_{2}=0\right)$, $\left(v_{1}+v_{2}\right),{\left(v_{1}\right)}^{2}+{\left(v_{2}\right)}^{2})$, expressing the physical assumption that the gas particles are point particles and that the particle trajectories in the collision are determined by Hamilton’s mechanics, but their details are ignored; only the momentum and the energy conservations are honored. In the general setting (48), we assume that the mappings are one-to-one and that their invariants are:

(49) $$B=\{b_{1}\left(1,\ldots ,n\right),\ldots ,b_{m}\left(1,\ldots ,n\right)\}$$

where *m* functions $\left(b_{1},\ldots ,b_{m}\right)$, satisfy:

(50) $$b_{1}\left(1,\ldots ,n\right)=b_{1}\left(\Upsilon \left(1,\ldots ,n\right)\right),\ldots ,b_{m}\left(1,\ldots ,n\right)=b_{m}\left(\Upsilon \left(1,\ldots ,n\right)\right)$$

Still following Boltzmann’s analysis, we introduce the thermodynamic forces:

(51) $$X\left(f^{*}\right)=f^{*}\left(1,\ldots ,n\right)-f^{*}\left(1^{\prime },\ldots ,n^{\prime }\right)$$

and the dissipation potential $\Xi^{↑}$. We choose $\Xi^{↑}$ to be the same as the one appearing in (41), but with *X* given in (51). We now add to the right-hand side of the Liouville Equation (47) an additional term $\Xi_{f^{*}}$. The resulting equation:

(52) $$\frac{\partial f}{\partial t}==\frac{\partial }{\partial r_{\alpha i}}\left(f\frac{\partial E_{f}}{\partial v_{\alpha i}}\right)+\frac{\partial }{\partial v_{\alpha i}}\left(f\frac{\partial E_{f}}{\partial r_{\alpha i}}\right)+\Xi_{f^{*}}$$

possesses the GENERIC structure, and consequently (see Section 2.1.3), its solutions approach the solutions to X=0. Such solutions form a manifold $M^{↑(eq)}=\left\{f\in M^{↑}|f^{*}=\sum_{i=1}^{m}<b_{i}^{*},b_{i}>\right\}$, parametrized by $b_{1}^{*},\ldots ,b_{m}^{*}$. With the Gibbs entropy, the dissipation potential Ξ given in (41), and the thermodynamic force *X* (51), the time-evolution Equation (52) becomes:

(53) $$\begin{array}{ccc} \frac{\partial f}{\partial t} & = & -\frac{\partial }{\partial r_{\alpha i}}\left(f\frac{\partial E_{f}}{\partial v_{\alpha i}}\right)+\frac{\partial }{\partial v_{\alpha i}}\left(f\frac{\partial E_{f}}{\partial r_{\alpha i}}\right)\\ & & +\int d1\ldots \int dnW\left(1,\ldots ,n,1^{\prime },\ldots ,n^{\prime }\right)\left(f\left(1^{\prime },\ldots ,n^{\prime }\right)-f\left(1,\ldots ,n\right)\right) \end{array}$$

where *W* is symmetric with respect to $\left(1,\ldots ,n\right)\to \left(1^{\prime },\ldots ,n^{\prime }\right)$, W≥0, and W=0 unless (50) holds. As is the case with the Liouville Equation (47), the complex and typically very nonlinear transformations Υ in the Boltzmann regularization mappings turn in the Liouville lift into a linear collision-like term.

The Boltzmann-inspired “retouch” of the phase portrait that we presented above is similar to the Ehrenfest regularization (Ehrenfest “retouch”) [21,56] in which very small pieces of trajectories are pre-processed.

A likely scenario of the Gibbs time-evolution passage to the equilibrium level is the following. The time-evolution begins with a weak dissipation, i.e., with a large set (49) of invariants, which means that only a few details are being ignored. In the course of the time-evolution, the dissipation increases due to the Grad–Villani enhancement (see Section 2.1.3) until the set B of invariants of the Boltzmann regularization mappings (48) becomes the small set $\left\{f\in M^{↑}|E\left(f\right)=E;N\left(f\right)=N\right\}$. In order to continue to discuss this scenario, one must discuss in particular the set of invariants (49) representing the seed of the dissipation, the applicability of the Grad–Villani enhancement of the dissipation, and the entropy entering the relation between *f* and $f^{*}$. All these discussions have to arise in an analysis of the solutions to (47).

### 4.3. Euler Fluid Mechanics: Local Conservation Laws

The level of fluid mechanics is the oldest [57] and undoubtedly the most important (at least from the application point of view) mesoscopic level. It has also served as a nucleus of other nearby levels, like for instance the level of the mechanics of solids, the level of the mechanics of complex fluids (rheology), classical nonequilibrium thermodynamics, and also many fields in mathematics. We recall below some aspects of its relations to mechanics, kinetic theory, and equilibrium thermodynamics. We also recall some branches of physics and mathematics that grew out of these investigations.

#### 4.3.1. Relation to Newton’s Mechanics

Leonhard Euler [57] introduced fluid mechanics as a continuum version of Newton’s mechanics of particles. The state variables are the fields:

(54) (ρ(r),e(r),u(r))

of mass, energy, and momentum, respectively. The total mass $M^{\left(fm\right)}=\int dr\rho \left(r\right)$, the total energy $E^{\left(fm\right)}=\int dre\left(r\right)$, and the total momentum $U^{\left(fm\right)}=\int dru\left(r\right)$ remain unchanged during the time-evolution. If we limit ourselves to fluids with only local interactions, then this property implies that the time-evolution equations form a system of local conservation laws (also called balance laws):

(55) $$\begin{array}{ccc} \frac{\partial \rho }{\partial t} & = & -\frac{\partial J_{i}^{\left(\rho \right)}}{\partial r_{i}}\\ \frac{\partial e}{\partial t} & = & -\frac{\partial J_{i}^{\left(e\right)}}{\partial r_{i}}\\ \frac{\partial u_{i}}{\partial t} & = & -\frac{\partial J_{ij}^{\left(u\right)}}{\partial r_{j}} \end{array}$$

The fields $\left(J^{\left(\rho \right)},J^{\left(e\right)},J^{\left(u\right)}\right)$ appearing in (55) are fluxes. Their specification as functions of the state variables (ρ(r),e(r),u(r)) is called a constitutive relation (see, e.g., [58,59]). The individual nature of the fluids is expressed in (55) in the constitutive relations. The third equation in (55) has two physical interpretations, one as a local conservation law (momentum conservation) and the other as a continuum version of Newton’s law (mass times acceleration equals force).

The Hamilton formulation of the governing equations of fluid mechanics appeared in 1859 in [30]. We present it in the form introduced by Arnold [31]. We begin with only the field u(r) in the set of the state variables (54). Our objective is to find a particular realization of (3) with x=u(r). In order to find the kinematics of u(r) (i.e., in order to determine the Poisson bivector $L^{↑}$), we turn to the physics of continuum. Following Euler [57], continuum is the space $R^{3}$, and its motion is a Lie group of transformations $R^{3}\to R^{3}$. Arnold [31] realized that the momentum field u(r) is an element of the dual of the Lie algebra that is associated with the Lie group of the transformations $R^{3}\to R^{3}$ and consequently that the Poisson bracket that is canonically associated with the Lie algebra [60,61] (which in the case of Lie group of transformations $R^{3}\to R^{3}$ has the form $\left\{A,B\right\}=\int dru_{i}\left(\frac{\partial A_{u_{i}}}{\partial r_{j}}B_{u_{j}}-\frac{\partial B_{u_{i}}}{\partial r_{j}}A_{u_{j}}\right)$ expresses mathematically the kinematics of the continuum.

In order to identify the kinematics of the full set (54) of the state variables that also satisfy the degeneracy requirement (see Section 2.1.1), we make an extra hypothesis about the time-evolution at the level of fluid mechanics. We replace the energy field e(r) in (54) with another scalar field s(r)=s(ρ,e,u;r) that is required to satisfy:

(56) $$\begin{array}{ccc} & & (\rho (r),e(r),u(r))⇆(\rho (r),s(r),u(r))\;\;\mathrm{is}\;\;a\;\;\mathrm{one}-\mathrm{to}-\mathrm{one}\;\;\mathrm{transformation}\\ & & \frac{\partial s}{\partial t}=s_{\rho }\frac{\partial \rho }{\partial t}+s_{e}\frac{\partial e}{\partial t}+s_{u_{i}}\frac{\partial u_{i}}{\partial t}=-\frac{\partial J_{i}^{\left(s\right)}}{\partial r_{i}} \end{array}$$

where $J^{\left(s\right)}$ is a flux of the field *s*. The flux $J^{\left(s\right)}$ is a function of the hydrodynamic fields. Its specification is a part of the constitutive relation. The physical interpretation of (56) will appear in Section 4.3.3 in the discussion of the relation of fluid mechanics with equilibrium thermodynamics. In the rest of this section, we shall use the fields (ρ(r),s(r),u(r)) as the state variables of fluid mechanics.

The two scalar fields (ρ(r),s(r)) are assumed to be passively advected with the motion of the continuum. With the use of the concept of the semi-direct product [60,61], the complete Poisson bracket expressing the kinematics of (ρ(r),s(r),u(r)) is given by:

(57) $$\begin{array}{ccc} \left\{A,B\right\} & = & \int dr\left[u_{i}\left(\frac{\partial A_{u_{i}}}{\partial r_{j}}B_{u_{j}}-\frac{\partial B_{u_{i}}}{\partial r_{j}}A_{u_{j}}\right)\right.\\ & & \left.+\rho \left(\frac{\partial A_{\rho }}{\partial r_{j}}B_{u_{j}}-\frac{\partial B_{\rho }}{\partial r_{j}}A_{u_{j}}\right)\right.\\ & & \left.s\left(\frac{\partial A_{s}}{\partial r_{j}}B_{u_{j}}-\frac{\partial B_{s}}{\partial r_{j}}A_{u_{j}}\right)\right] \end{array}$$

The equations (3) governing the Hamiltonian time-evolution of (ρ(r),s(r),u(r)) are thus:

(58) $$\begin{array}{ccc} \frac{\partial \rho }{\partial t} & = & -\frac{\partial J_{i}^{\left(\rho \right)}}{\partial r_{i}}\\ \frac{\partial s}{\partial t} & = & -\frac{\partial J_{i}^{\left(s\right)}}{\partial r_{i}}\\ \frac{\partial u_{i}}{\partial t} & = & -\frac{\partial J_{ij}^{\left(u\right)}}{\partial r_{j}} \end{array}$$

where:

(59) $$\begin{array}{ccc} J_{i}^{\left(\rho \right)} & = & \rho E_{u_{i}}^{↑}\\ J_{i}^{\left(s\right)} & = & sE_{u_{i}}^{↑}\\ J_{ij}^{\left(u\right)} & = & u_{i}E_{u_{j}}^{↑}+p\delta_{ij}\\ p & = & -e+\rho E_{\rho }^{↑}+sE_{s}^{↑}+u_{i}E_{u_{i}}^{↑} \end{array}$$

We see thus that the requirement expressed in the second equation in (56) is satisfied, and thus, S=∫drs(r) is the Casimir of the Poisson bracket (57).

The Hamiltonian formulation of Euler’s equations (58) has at least four advantages: (i) the constitutive relation for the non-dissipative part of the time-evolution is specified (see (59)) with only the energy E(ρ,s,u) remaining to be determined; (ii) it provides a framework for investigations into the dynamics of more general fluids (e.g., complex fluids studied in rheology [2]) for which the framework (55) of balance laws cannot be used; (iii) it can also be used at other mesoscopic and microscopic levels of description; (iv) it offers promising new approaches to numerical fluid mechanics [62]. In spite of these obvious advantages, the Hamiltonian formulation is still absent in most standard textbooks of fluid mechanics.

The gradient part of the time-evolution appears in Section 4.3.3 in the discussion of the relation between the level of fluid mechanics and the equilibrium level.

Looking at (55) and (56) just from the mathematical point of view, we see a system of local conservation laws (55) implying another companion conservation law (56). Are there any mathematical consequences of the physical regularity of (55) expressed in the requirement (56)? Godunov [63,64] (see also [65,66,67,68]) showed that the physical regularity implies the mathematical regularity in the sense that (56) guarantees that the Riemannian problem for (58) is well posed. More specifically, (56) implies that (55) rewritten in the conjugate state variables is a system of symmetric local conservation laws.

The observations that (55), besides being the system of local conservation laws, is also a Hamiltonian system and that (56) is a stronger formulation of the degeneracy of the Hamiltonian structure (∫drs(r) is a Casimir) evoke several questions that remain unanswered. For instance: (i) When does a general system of local conservation laws possess the Hamiltonian structure? (ii) Does the degeneracy of a Hamiltonian system imply an increase in its mathematical regularity?

#### 4.3.2. Relation to More Microscopic Levels

The level of fluid mechanics is presented in the previous section as a continuum version of Newton’s (or Hamilton’s) dynamics. Let us now take an upper mesoscopic level L that involves more details than the level of fluid mechanics (e.g., the level of kinetic theory) and consider the passage L→ fluid mechanics. We have already recalled one such passage with L being the level of kinetic theory in Section 4.3.2. An alternative way (a way based on the hierarchy formulation of the Boltzmann equation) to make the same passage is discussed below in Section 5.

With the microscopic level (i.e., a level at which an *n* particle distribution function, $n∼10^{23}$, rather than a one particle distribution function serves as the state variable) playing the role of the upper level L, the passage L→ fluid mechanics was investigated by Kirkwood [69,70]. This type of investigation has led to the theoretical fluid mechanics of complex fluids as for example polymeric fluids and suspensions [71].

We make two remarks. First, we note an important difference between the multiscale viewpoint of the passage Boltzmann kinetic equation → fluid mechanics and its classical analysis found for example in [18,72,73,74,75,76]. In the latter, the Boltzmann kinetic equation plays the role of a microscopic basis for the classical nonequilibrium thermodynamics. In the former, the Boltzmann kinetic theory, as well as the classical nonequilibrium thermodynamics are two particular realizations, at two different levels, of a single, but abstract nonequilibrium thermodynamics.

In the second remark, we note an obvious paradox in the investigation of Boltzmann kinetic equation → fluid mechanics. Boltzmann’s kinetic theory is applicable only to ideal gases, while the domain of applicability of fluid mechanics includes a large family of fluids. The usefulness of the investigation Boltzmann kinetic equation → fluid mechanics is an indirect proof of the usefulness of seeing mesoscopic dynamical systems in a modular way as is done for example in Section 2.1. What transpires from kinetic theory to fluid mechanics are only some of its modules (in particular, the overall mathematical structure), not the complete theory (in particular, not specific energies and specific entropies). The completely straightforward and completely rigorous derivation of the Poisson bracket expressing the kinematics of the hydrodynamic state variables from the Poisson bracket expressing the kinematics of the one particle distribution function, which we recalled at the end of Section 4.1, illustrates this point well. As we shall see also in the next section, some modules of the mathematical structure of the Boltzmann kinetic equation that are revealed in its Grad hierarchy formulation have inspired, and continue to inspire, not only classical fluid mechanics, but also its extensions towards dense fluids, polymeric fluids, and many other types of complex fluids.

#### 4.3.3. Relation to Equilibrium Thermodynamics: Nonequilibrium Thermodynamics

Inquiries into relations between fluid mechanics and equilibrium thermodynamics gave rise to nonequilibrium thermodynamics. Our objective in this section is to identify in its tumultuous history a path pointing to multiscale thermodynamics discussed in this paper. We present the path as a sequence of four steps.

The basis on which classical nonequilibrium thermodynamics stands is the continuum mechanics introduced by Euler and Bernoulli [57]. If we put it into the context of Section 4.3.1, it is the fluid mechanics with only the momentum field u(r) playing the role of the state variable. In other words, the fluids under investigation are isothermal and incompressible. This type of fluid mechanics has played and continues to play an enormously important role in all types of the most basic, as well as the most advanced technologies.

The first step towards multiscale thermodynamics is the mechanics of non-isothermal and compressible fluids and the investigations of its compatibility with classical equilibrium thermodynamics. Two extra fields, namely the fields of mass density and internal energy, are adopted for the set of state variables. In such an enlarged setting, the fluid mechanics becomes essentially a local classical thermodynamics superimposed on the mechanics of continuum. The equilibrium fundamental thermodynamic relation becomes a local equilibrium fundamental thermodynamic relation, and the entropy conservation takes the form of the local conservation law (56). The Navier–Stokes friction and the Fourier heat diffusion enter the entropy production (or the dissipation potential), which with the entropy are two potentials of non-mechanical origin that join the formulation of fluid mechanics. We note that the last equation in (59) in which the local pressure p(r) is expressed in terms of the hydrodynamic fields (ρ(r),u(r),s(r)) and the energy e(ρ,u,s;r) is the same (in the absence of the flow, i.e., if u≡0) as the expression for the equilibrium pressure in equilibrium thermodynamics. This means that the requirement of the Hamiltonian structure of the non-dissipative time-evolution of the hydrodynamic fields (ρ(r),u(r),s(r)) together with the requirement of the conservation of the total momentum ∫dru(r) is equivalent to a part of the local equilibrium assumption. A complete equivalence still requires an additional requirement. $E_{s}^{↑}$ has to be interpreted as the local absolute temperature. This extra requirement then also means (due to the positivity of the absolute temperature) that fluid mechanics can be cast into two equivalent representations: (i) energy representation with the state variables (ρ(r),u(r),s(r)) and the fundamental thermodynamic relation e(r)=e(ρ,u,s) and (ii) entropy representation with the state variables (ρ(r),u(r),e(r)) and the fundamental thermodynamic relation s(r)=e(ρ,u,e). As we have seen in Section 2.2, this type of a two representation formulation does not extend to more microscopic levels.

Combinations of mechanics and equilibrium thermodynamics inspired also more abstract viewpoints. Their explorations constitute the second step in the evolution path of the nonequilibrium thermodynamics. The first example of an abstraction inspired by fluid mechanics is the replacement of (55) with a general system of local conservation laws governing the time-evolution of *n* fields $\left(\xi_{1}\left(r\right),\ldots ,\xi_{n}\left(r\right)\right)$ with an extra companion local conservation law governing the time-evolution of the (n+1)th field $\xi_{n+1}\left(r\right)$ that is a convex function of $\left(\xi_{1}\left(r\right),\ldots ,\xi_{n}\left(r\right)\right)$. From the physical point of view, the (n+1)th field is the entropy field (see the end of Section 4.3.1).

The second example of the abstraction is the emergence (already in the early stages of the development of nonequilibrium thermodynamics [77,78,79]) of the concepts of entropy production, thermodynamic forces, and thermodynamic fluxes [80]. Their particular realizations in the context of fluid mechanics have served as their illustrations, but they were seen from the beginning as abstract concepts. The thermodynamic fluxes and thermodynamic forces together form the entropy production:

(60) S=<X,J>

or in terms of the dissipation potential Ξ(X):

(61) $$S=<X,\Xi_{X}>$$

Particularly significant in this line of research are the results of Onsager [81], who showed that in the case of the quadratic dissipation potential Ξ=<X,ΛX> (which physically corresponds to situations with small *X* and thus situations when the macroscopic systems under investigation are close to equilibrium), the operator Λ is symmetric and positive definite. The rate thermodynamics that we recalled in Section 3 is an incorporation of this type of investigations into the larger context of multiscale thermodynamics.

The third example is Truesdell’s axiomatic formulation of continuum mechanics [58]. While his choice of axioms may be questioned, the emphasis on the abstract mathematics is unquestionably a significant contribution to fluid mechanics. For instance, restrictions on the choice of constitutive relations brought about by the requirement of the entropy increase were first investigated in Truesdell’s formulation of fluid mechanics [82].

In the spirit of multiscale thermodynamics discussed in this paper, an important criterion for abstract formulations is the occurrence and applicability at all levels. Not all Truesdell’s axioms fulfill this criterion. For instance, the local temperature cannot be seen at more microscopic levels as a fundamental state variable (see Section 2.2).

The third step on the path to multiscale thermodynamics is seeing Boltzmann’s kinetic theory as nonequilibrium thermodynamics itself, not only as a microscopic basis for classical (i.e., fluid mechanics-based) nonequilibrium thermodynamics.

The fourth step towards multiscale thermodynamics is the necessity to enlarge the set of the five hydrodynamic fields playing the role of state variables in classical fluid mechanics when dealing with complex fluids (as for example polymeric fluids and suspensions). The molecules (or alternatively, particles in suspensions) composing the complex fluids deform and reorient themselves at the same time scale as the hydrodynamic fields evolve. Consequently, extra fields characterizing the internal structure have to be adopted for the set of state variables. However, then, the system of local conservation laws (also called “balance laws”) (55) cannot be the point of departure (as in classical fluid mechanics and classical nonequilibrium thermodynamics) since the extra fields are typically not conserved. What is then an overall structure that would replace (55)? In the setting of mesoscopic thermodynamics, the answer is: it is the Hamiltonian (or the GENERIC) structure.

### 4.4. Guldberg–Waage Chemical Kinetics: Dissipation Potentials

Historically, the first investigation of the time-evolution passage, which we recalled in Section 4.1, is also historically the first introduction of the dissipation-potential gradient dynamics (7). Initially, the Boltzmann collision term did not have the form of the right-hand side of (7). Its dissipation-potential formulation became possible [33] only after an observation (made by Ludwig Waldmann in [45]) that binary collisions can be seen as chemical reactions and after the gradual realization [7,83,84,85,86] that the time-evolution arising in chemical kinetics can be cast into the form of (7). In this section, we argue that the chemical kinetics provides a particularly suitable setting for a deeper investigation of the solution to the GENERIC equation.

The need to extend linear relations between thermodynamic fluxes and thermodynamic forces to nonlinear relations appeared very clearly in particular in chemical kinetics [87] describing the time-evolution of chemically reacting species. Let us consider *q* chemical reactions among *p* components (we assume p>q):

(62) $$\alpha_{1j}A_{i}+\ldots +\alpha_{pj}A_{p}⇆\beta_{1j}A_{i}+\ldots +\beta_{pj}A_{p}$$

where $A_{1},\ldots ,A_{p}$ denote the species. The quantities:

(63) $$\gamma_{ij}=\alpha_{ij}-\beta_{ij};\;\;\;i=1,\ldots ,p;\;\;\;j=1,\ldots ,q$$

are called stoichiometric coefficients, and the matrix $\Gamma =(\gamma_{ij});\;\;\;i=1,\ldots ,p;\;\;\;j=1,\ldots ,q$ is called a stoichiometric matrix.

The state variables in isothermal chemical kinetics are $n=(n_{1},\ldots ,n_{p})$, denoting the number of moles of the species, and the constant temperature *T*. The equations:

(64) $${\dot{n}}_{i}=\gamma_{ij}J_{j}$$

governing the time-evolution of ***n*** resemble the system of local conservation laws (55). The gradient ∇ appearing in (55) is replaced by the stoichiometric matrix Γ, and the fluxes $\left(J_{1},\ldots ,J_{q}\right)$ in (64) are extensions of the reactions.

Guldberg and Waage [87] completed (64) with the mass-action-law constitutive relations:

(65) $$J_{j}={\vec{k}}_{j}n_{1}^{\alpha_{1j}}\ldots n_{p}^{\alpha_{pj}}-{\overset{\leftarrow }{k}}_{j}n_{1}^{\beta_{1j}}\ldots n_{p}^{\beta_{pj}};\;\;\;j=1,\ldots ,q$$

where ${\vec{k}}_{j},{\overset{\leftarrow }{k}}_{j}$ are the rate coefficients of the forward *j*-th reaction and the backward *j*-th reaction, respectively.

It has been gradually [7,46,83,84,85,86,88,89] realized that (64) with the constitutive relation (65) can be cast into the form:

(66) $${\dot{n}}_{i}=-\Xi_{n_{i}^{*}}\left(n,T,X\right)$$

with the dissipation potential Ξ given in (41) in which the thermodynamic forces $X=(X_{1},\ldots ,X_{q})$, called in chemical kinetics affinities, are:

(67) $$X_{j}=\gamma_{ji}n_{i}^{*};\;\;j=1,\ldots ,q$$

$n_{i}^{*}=\Phi_{n_{i}}\left(n,T\right)$. *W* is expressed in terms of the rate coefficients ${\vec{k}}_{j},{\overset{\leftarrow }{k}}_{j}$ (see details in [46]) and an appropriately chosen thermodynamic potential Φ(n,T) (see the details in [46]).

#### 4.4.1. GENERIC Formulation of Chemical Kinetics

The state spaces in the Guldberg–Waage dynamics are finite-dimensional. This is obviously an advantage in the investigation of solutions. Another advantage of the Guldberg–Waage dynamics, in particular in the context of multiscale thermodynamics, is the natural appearance of intermediate levels. We begin by extending (66) to a full GENERIC Equation (12), and the intermediate levels are discussed in Section 4.4.2 below.

From the physical point of view, the extension of (66) to GENERIC expresses mathematically an inclusion of inertia in chemical reactions. The fluxes $J=(J_{1},\ldots ,J_{q})$ are promoted to the status of independent state variables. We are thus making in chemical kinetics the same type of extension as the one made in fluid mechanics in [67,76]. The time-evolution of the fluxes is assumed to be faster than the time-evolution of ***n***. After the fast time-evolution of the fluxes is complete, the subsequent slow time-evolution of ***n*** is expected to be governed by the standard chemical kinetics equations (66).

Equations:

(68) $$\left(\begin{array}{c} \dot{n}\\ \dot{w} \end{array}\right)=\left(\begin{array}{cc} 0 & \Gamma \\ -\Gamma^{T} & 0 \end{array}\right)\left(\begin{array}{c} n^{⋆}\\ w^{⋆} \end{array}\right)-\left(\begin{array}{c} 0\\ \Theta_{w^{⋆}} \end{array}\right)$$

governing such a time-evolution of the extended set of state variables were introduced in [43,88]: The extra state variables $w=(w_{1},\ldots ,w_{q})$ are related to $J=(J_{1},\ldots ,J_{q})$ by $w_{j}^{⋆}=J_{j};j=1,\ldots ,q$, where $n^{⋆}=\Phi_{n}^{\left(ext\right)}$, $w^{⋆}=\Phi_{w}^{\left(ext\right)}$, $\Phi^{\left(ext\right)}\left(n,w\right)$ is the thermodynamic potential in the extended theory, and $\Theta (n^{⋆},w^{⋆})$ is the dissipation potential in the extended theory. First, we show that (68) is a particular realization of the GENERIC Equation (12), and then, we find the relation between the extended dissipation potential Θ appearing in the extended chemical kinetics (68) and the dissipation potential Ξ appearing in the standard chemical kinetics (66).

The first term on the right-hand side of (68) is Hamiltonian since $\left(A_{n},A_{w}\right)\left(\begin{array}{cc} 0 & \Gamma \\ -\Gamma^{T} & 0 \end{array}\right)$$\left(\begin{array}{c} B_{n}\\ B_{w} \end{array}\right)$ is a Poisson bracket (because the matrix $\left(\begin{array}{cc} 0 & \Gamma \\ -\Gamma^{T} & 0 \end{array}\right)$ is skew-symmetric and independent of the state variables), and *A* and *B* are real-valued sufficiently regular functions of the state variables. The time-evolution Equation (68) is thus a particular realization of (12).

Now, we turn to the problem of finding the relation between the dissipation potential Ξ appearing in (66) and the extended dissipation potential Θ appearing in (68). We assume that the time-evolution of *w* is faster than the time-evolution of ***n***. Moreover, we require that when the fast variable *w* reaches its stationary state, i.e., when:

(69) $$-\Gamma^{T}n^{⋆}-\Theta_{w^{⋆}}=0$$

then the subsequent time-evolution is governed by (66). This requirement is satisfied provided the dissipation potentials Ξ(X) and the extended dissipation potential Θ(Y) are related by:

(70) $$\Xi_{n^{*}}={\left[\Theta_{Y^{†}}^{†}\left(Y^{†}\right)\right]}_{Y^{†}=-X}$$

and the thermodynamic potential Φ(n,T) and the extended thermodynamic potential $\Phi^{\left(ext\right)}\left(n,w,T\right)$ are related in such a way that $n^{*}=n^{⋆}$.

The superscript † denotes the conjugation with respect to the dissipation potential Θ, i.e., ${\left(w^{⋆}\right)}^{†}=\Theta_{w^{⋆}}$. By the symbol *Y*, we denote the extended thermodynamic force $Y=\Gamma w^{⋆}$. The dissipation potential $\Theta^{†}\left(Y^{†},T\right)$ is the Legendre transformation of Θ(Y,T).

We now prove that (70) implies the compatibility between (66) and (68). We introduce the dissipation thermodynamic potential:

$$\Psi \left(Y,T;Y^{†}\right)=-\Theta \left(Y,T\right)+<Y^{†},Y>,$$

and recall that $Y=\Theta_{Y^{†}}^{†}$. Finally, we note that (69) is the equation ${\left[\Psi_{Y}\left(Y,T;Y^{†}\right)\right]}_{Y^{†}=-X}=0$.

Before leaving this section, we make a comment about the difference between the potentials like the energy, entropy, number of moles, and dissipation potential. Gradients of the former generate the forces driving the time-evolution, and gradients of the latter transform the forces (co-vectors) into vector fields. Specifically, the forces generated by the energy are transformed into the Hamiltonian vector fields by the Poisson bracket. The forces generated by the entropy are transformed into vector fields by the dissipation potential. The dissipation potential in gradient dynamics is thus a counterpart of the Poisson bracket in the Hamiltonian dynamics. Locally, in a small neighborhood of X=0, the dissipation potentials become quadratic. Such quadratic dissipation potentials can be then interpreted as a dissipation brackets (introduced in [33]). In this linearized formulation, there are thus two brackets transforming forces into vectors. The symmetric dissipation bracket can be extended to a nonlinear dissipation potential. No such extension can be made for the skew-symmetric Poisson bracket.

If both the Poisson bracket and the dissipation potentials participate in the dynamics, then the Poisson bracket is completely insensitive to the forces generated by the entropy and the total number of moles. The dissipation potential is on the other hand completely insensitive to the forces generated by the energy and the total number of moles. The insensitivities are mathematically expressed in the degeneracies of the Poisson brackets and the dissipation potentials.

In the contact structure formulation of the GENERIC dynamics (see Section 2.3), both the Poisson bracket and the dissipation potential become potentials generating the contact-structure preserving time-evolution. The potentials like the energy, the entropy, and number of moles determine the Legendre submanifold on which the time-evolution takes place. Dissipation potentials arise [90] also in the stochastic thermodynamics in extensions to the large deviation stochastic theory.

#### 4.4.2. Problem: Grad–Villani Dissipation Enhancement in Chemical Kinetics

Let some of the chemical reactions (62) be faster than the others. Specifically, let the first *m* reactions be fast and the remaining q−m reactions slow. We write the stoichiometric matrix in the form:

(71) $$\Gamma =\Gamma^{\left(fast\right)}+\Gamma^{\left(slow\right)}$$

The matrix $\Gamma^{\left(fast\right)}=\left(\gamma_{ij}^{\left(fast\right)}=\gamma_{ij}\right)$, i=1,…,p; j=1,…,m and $\left(\gamma^{\left(fast\right)}=0\right)$, i=1,…,p; j=m+1,…,q. The matrix $\Gamma^{\left(slow\right)}=\left(\gamma_{ij}^{\left(slow\right)}=\gamma_{ij}\right)$i=1,…,p, j=m+1,…,q and $\left(\gamma^{\left(slow\right)}=0\right)$, i=1,…,p; j=1,…,m. The split (68) induces (see (66)) the splits $X=X^{\left(fast\right)}+X^{\left(slow\right)}$ and $Y=Y^{\left(fast\right)}+Y^{\left(slow\right)}$. The passage to the chemical equilibrium at which X=0 can be seen as a two stage process. The first stage is the approach to the fast equilibrium at which $X^{\left(fast\right)}=0$. The fast approach is then followed by a slow time-evolution that terminates at the total equilibrium at which both $X^{\left(fast\right)}$ and $X^{\left(slow\right)}$ equal zero. This type of time-evolution was recently investigated in the setting of the stochastic thermodynamics in [91]. With the results obtained in the preceding section, we can investigate the same problem in the setting of multiscale thermodynamics. In this paper, we limit ourselves only to formulating two questions.

Question 1:

How exactly are solutions to (66) and solutions to (68) related (the dissipation potential Θ appearing in (68) is assumed to satisfy (70))?

Question 2:

We modify Equation (68) by replacing the dissipation potential Θ with the fast dissipation potential $\Theta^{\left(fast\right)}={\left[\Theta \right]}_{Y=Y^{\left(fast\right)}}$ that provides a weaker dissipation. Do solutions to such modified equations with a weaker dissipation still approach the same chemical equilibrium state as solutions to (68)? Investigations of this question are investigations of the Grad–Villani dissipation enhancement (see Section 2.1.3) in the context of chemical kinetics.

### 4.5. Statistical Mechanics

The problems discussed in this and the previous sections belong to statistical mechanics. The way we presented and discussed microscopic, mesoscopic, and macroscopic dynamics suggests an alternative view of statistical mechanics. First, we recall the conventional viewpoint, and then, we introduce its alternative.

Traditionally, statistical mechanics is divided into two parts: equilibrium and nonequilibrium. In the broader viewpoint of the microscopic-mesoscopic-macroscopic dynamics that we are taking in this paper, thermodynamics has to be also seen as a part of statistical mechanics. Thermodynamics traditionally is divided into classical equilibrium and nonequilibrium. In the following four paragraphs, we briefly recall the main tenets of the four parts of statistical mechanics.

Equilibrium statistical mechanics is an investigation of the MaxEnt passage from the level of classical (or quantum) mechanics of ∼10${}^{23}$ particles to the equilibrium level with the volume *V*, number of moles *N*, and energy *E* playing the role of the state variables (see Section 2.2 with the upper thermodynamic relation (45)). The investigation includes its thermodynamic limit N→∞,V→∞,N/V=const. in [92], as well as its various geometrical deformations (e.g., in [93]) and approximations [94].

Nonequilibrium statistical mechanics is a collection of various investigations, both in deterministic and stochastic settings, of the time evolution taking place at mesoscopic levels [95]. The collection has no clear organizing principle.

Classical equilibrium thermodynamics [1,8] is a very clearly formulated theory. However, the postulates on which it stands are disconnected from the more microscopic viewpoints, and its domain of applicability is limited to macroscopic systems at equilibrium, i.e., to macroscopic systems that are particularly prepared by leaving them a sufficiently long time without external influences.

Nonequilibrium thermodynamics is a theory recalled in Section 4.3.3. The difference between nonequilibrium statistical mechanics and nonequilibrium thermodynamics is only in the state variables. If the state variables can be physically interpreted as distribution functions, then it is customary to consider the investigation as a part of statistical mechanics. For example, if the state variable is the one particle distribution function, then investigations of its time-evolution belong traditionally to kinetic theory, which is considered to be a part of nonequilibrium statistical mechanics. If however the state variables are the first five moments of the one particle distribution function (i.e., the hydrodynamic fields), then investigations of their time-evolution belong traditionally to nonequilibrium thermodynamics.

Following Section 2, we suggest regarding statistical mechanics (which we call multiscale thermodynamics) as an oriented graph in which vertices are levels and links are reductions. The links point from the upper to lower levels. Depending on the features of the time-evolution that are being compared in the reductions, the links are of three types: state space, rate, and boundary. Each link is then of two types: time-evolution and MaxEnt. Altogether, every upper level is connected with a lower level by three pairs of arrows, all pointing to the lower level.

State space reductions are described in Section 2. The essence of the reduction is a recognition of a pattern in the upper phase portrait. The recognized pattern is then identified with the lower phase portrait. We recall that an upper phase portrait is a collection of trajectories generated by an ensemble of upper vector fields and passing through all points in the upper state space. Similarly, the lower phase portrait is a collection of trajectories generated by an ensemble of lower vector fields and passing through all points in the lower state space.

Rate reductions are described in Section 3. They are the same as the state space reductions except that the pattern is searched in rate phase portraits rather than in phase portraits. The rate phase portrait is a collection of rate trajectories in the space of vector fields on the state space. The rate trajectories are solutions of the time-evolution lifted from the state space to the space of vector fields on the state space.

Boundary reductions are the same type of reductions as the two previous ones except that the focus is put on the behavior in the region in which the bulk meets the boundary of the system under investigation. In this paper, we have not discussed them, but this part of statistical mechanics is obviously very important and will be pursued in the future.

All three types of reductions are made by following reducing time-evolutions to their conclusions. All reducing time-evolutions are generated by a potential called an entropy. Two different reductions are generated (at least in general) by two different entropies. The entropy increases during the reducing time-evolution. The states at which the entropy reaches its maximum (subject to constraints determined by the target lower level) are thus the states representing the lower state variables in the upper state space. These states can be thus reached either by following the reducing time-evolution to its conclusion or simply by maximizing the entropy subject to appropriate constraints. The former way is the time-evolution reduction (see Section 2.1), and the latter is the MaxEnt reduction (see Section 2.2).

Two vertices in the multiscale-thermodynamics graphs have a special status. One is with no incoming arrows and the other with no outgoing arrows. The former is the most microscopic level at which macroscopic systems are seen as composed of ∼10${}^{23}$ particles. The latter is the level of the classical equilibrium thermodynamics on which states are characterized by the volume, the number of moles, and the energy.

The reducing entropies at the most microscopic level are potentials, called Casimirs, that remain unchanged during the upper time-evolution. There are infinitely many Casimirs. In order to single out one among them, we need to identify a nucleus of dissipation (e.g., collisions in dilute gases). The nucleus increases during the time-evolution, and the resulting dissipative time-evolution gives rise to a reduction represented by an outgoing arrow. The reduction is generated by a potential, which is then the entropy singled out among the infinitely many Casimirs.

The reduced entropies appearing at the level of the classical equilibrium thermodynamics due to incoming arrows manifest themselves in the separation of the total energy into a macroscopic mechanical energy and heat. The former can be directly and completely converted into macroscopic mechanical work, the latter only incompletely in Carnot’s machines.

Seeing the conventional viewpoint of statistical mechanics in the context of the graph of multiscale thermodynamics, we note that the conventional viewpoint concentrates on some particular links (in particular, those leaving the microscopic level), while the graph viewpoint puts the same attention on all links in the graph. We may anticipate that a general theory of the graph (in particular, the identification of the common features of all the links) can contribute to a deeper understanding of the links leaving the microscopic level (which are obviously of particular importance for understanding emerging phenomena). For instance, it was suggested in Section 4.2 that the Grad–Villani dissipation enhancement that seems to be present in all links may be such a contribution.

## 5. Kinematics-Preserving Hierarchies

This section illustrates the use of multiscale thermodynamics in advancing specific problems. One of the oldest and probably still the most frequently used strategies to make reductions is to begin by casting the upper level governing equations into a hierarchy of equations. The hierarchy reformulation is chosen in such a way that the lower part of the hierarchy is already the system of reduced governing equations that we look for except that the equations still remain coupled to the rest of the hierarchy. Our objective in this section is to place the hierarchy reductions into the larger context of multiscale thermodynamics and to show some of the implications.

We present the mathematical formulation first for the case when the upper state variable $x\in M^{↑}$ is the N particle distribution function f(1,…,N) (we recall that we use the shorthand notation $1=\left(r_{1},v_{1}\right),\ldots ,N=\left(r_{N},v_{N}\right)$). Our objective is to lift the time-evolution of *f* to space $M^{↑}$ with the state variables $Z=\left(Z,f\left(1,\ldots ,N\right)\right)\in M^{↑}$, where:

(72) $$\begin{array}{ccc} Z=(Z_{1},\ldots ,Z_{n}) & = & \left(\int d1\ldots \int dNf\left(1,\ldots ,N\right)z_{1}\left(1,\ldots N\right),\ldots ,\right.\\ & & \left.\int d1\ldots \int dNf\left(1,\ldots ,N\right)z_{n}\left(1,\ldots N\right)\right) \end{array}$$

and $z\left(1,\ldots ,N\right)=(z_{1}\left(1,\ldots ,N\right),\ldots ,z_{n}\left(1,\ldots ,N\right))$ is a fixed set of *n* functions. We recall that reduction is a pattern recognition in the upper phase portrait. We assume that from some previous considerations, we already have a reason to anticipate that Z will play an important role in expressing the pattern (see the examples in Section 5.2.1 and Section 5.2.2).

As for the time-evolution of *f* at the level L, we restrict ourselves to the time-evolution equations in the form of (3). In other words, we consider the time-evolution equations in the form:

(73) $$\dot{A}={\left\{A,E\right\}}^{↑}\;\;\;\;\forall A$$

where ${\left\{A,B\right\}}^{↑}$ is a Poisson bracket. We thus consider in this section only Hamiltonian dynamics. However, the contact geometry setting that is discussed in Section 2.3, a slightly modified Equation (73) (see Equation (7.7) in [43]), represents also GENERIC dynamics. The kinematics-preserving hierarchy formulation of GENERIC dynamics will be explored in a future paper.

Having the time-evolution Equation (3) and the mapping (72), the first equation in the standard hierarchy reformulation of (3) is obtained by multiplying (3) by $z_{1}\left(1,\ldots ,N\right)$ and integrating over ∫d1…∫dN. The second equation is obtained in the same way, but with $z_{2}\left(1,\ldots ,N\right)$ replacing $z_{1}\left(1,\ldots ,N\right)$. Continuing this process, we obtain the standard hierarchy consisting of n+1 time-evolution equations; *n* equations governing the time-evolution of Z that are coupled to the (n+1)th Equation (47) governing the time-evolution of *f*. The next step in the reduction is the “closure of the hierarchy” consisting of expressing *f* in terms of Z. The final reduced dynamics in $M^{↓}$ consists of *n* equations governing the time-evolution of Z. In the unclosed form, the hierarchical reformulation represents in fact a coupled dynamics of the upper and the lower levels. By choosing appropriately the functions $z\left(1,\ldots ,N\right)=(z_{1}\left(1,\ldots ,N\right),\ldots ,z_{n}\left(1,\ldots ,N\right))$ (see, e.g., the illustrations in Section 5.2.1 and Section 5.2.2), the resulting hierarchy can be made to involve only Z and not *f*. The prize to pay for such elimination of the overall state variable *f* is that n=∞, i.e., the hierarchy is infinite. The closure in such a case consists of replacing an infinite hierarchy with a finite hierarchy (see Section 5.2.1 and Section 5.2.2 below).

In this paper, we take another path. We shall make an alternative hierarchy reformulation of (3). We use the fact that the vector field (3) (i.e., the right-hand side of (3)) is composed of two structural elements, and only one of them is cast into the hierarchy form. The vector field (3) is a force (the gradient of a potential *E*) transformed into a vector by kinematics expressed mathematically in the bivector $L^{↑}$ (or equivalently in the Poisson bracket ${\left\{,\right\}}^{↑}$). In the hierarchy reformulation, we concentrate only on the kinematics, i.e., only on the Poisson bracket ${\left\{,\right\}}^{↑}$. We reformulate it into a hierarchy form that retains the Poisson structure. The energy *E* remains in the reformulation undetermined. Since it is the energy where the individual nature of the macroscopic systems is expressed, the particular physics of the system under investigation does not enter the mathematical modeling before starting the hierarchy reformulation (as is done in the standard approach), but after the kinematics-preserving hierarchy reformulation has been made. The specification of the energy becomes thus an extra tool in reductions.

In order to obtain such a kinematics-preserving reformulation of the Liouville Equation (47), we proceed as follows. The functions *A* and *B* in the Poisson bracket {A,B} are assumed to depend on *f* both directly and through their dependence on Z given in (72). This means that $A_{f(1,\ldots ,N)}$ turns into $z_{\alpha }\left(1,\ldots ,N\right)A_{Z_{\alpha }}+A_{f}$, where α=1,…,n (the summation convention over repeated indices is used). With these expressions for $A_{f}$ and $B_{f}$, the Poisson bracket (46) becomes:

(74) $$\begin{array}{ccc} \left\{A,B\right\} & = & \int d1\ldots \int dNf\left[\frac{\partial z_{\alpha }}{\partial r_{\gamma i}}\frac{\partial z_{\beta }}{\partial v_{\gamma i}}\left(A_{Z_{\alpha }}B_{Z_{\beta }}-B_{Z_{\alpha }}A_{Z_{\beta }}\right)\right.\\ & & \left.+\frac{\partial z_{\alpha }}{\partial r_{\gamma i}}\left(A_{Z_{\alpha }}\frac{\partial B_{f}}{\partial v_{\gamma i}}-B_{Z_{\alpha }}\frac{\partial A_{f}}{\partial v_{\gamma i}}\right)\right]\\ & & \left.+\frac{\partial z_{\alpha }}{\partial v_{\gamma i}}\left(B_{Z_{\alpha }}\frac{\partial A_{f}}{\partial r_{\gamma i}}-A_{Z_{\alpha }}\frac{\partial B_{f}}{\partial r_{\gamma i}}\right)\right.\\ & & \left.+\left(\frac{\partial A_{f}}{\partial r_{\gamma i}}\frac{\partial B_{f}}{\partial v_{\gamma i}}-\frac{\partial B_{f}}{\partial r_{\gamma i}}\frac{\partial A_{f}}{\partial v_{\gamma i}}\right)\right] \end{array}$$

The time-evolution equations (73) with ${\left\{A,B\right\}}^{↑}$ given in (71) take the form:

(75) $$\begin{array}{ccc} {\dot{Z}}_{\alpha } & = & \int d1\ldots \int dNf\left[\left(\frac{\partial z_{\alpha }}{\partial r_{\gamma i}}\frac{\partial z_{\beta }}{\partial v_{\gamma i}}-\frac{\partial z_{\beta }}{\partial r_{\gamma i}}\frac{\partial z_{\alpha }}{\partial v_{\gamma i}}\right)E_{Z_{\beta }}\right.\\ & & \left.+\left(\frac{\partial z_{\alpha }}{\partial r_{\gamma i}}\frac{\partial E_{f}}{\partial v_{\gamma i}}-\frac{\partial z_{\alpha }}{\partial v_{\gamma i}}\frac{\partial E_{f}}{\partial r_{\gamma i}}\right)\right]\\ \frac{\partial f}{\partial t} & = & -\frac{\partial }{\partial r_{\gamma i}}\left(f\frac{\partial z_{\alpha }}{\partial v_{\gamma i}}\right)E_{Z_{\alpha }}+\frac{\partial }{\partial v_{\gamma i}}\left(f\frac{\partial z_{\alpha }}{\partial r_{\gamma i}}\right)E_{Z_{\alpha }}\\ & & -\frac{\partial }{\partial r_{\gamma i}}\left(f\frac{\partial E_{f}}{\partial v_{\gamma i}}\right)+\frac{\partial }{\partial v_{\gamma i}}\left(f\frac{\partial E_{f}}{\partial r_{\gamma i}}\right) \end{array}$$

Summing up, we have cast (47) into the form (75). Both Equations (47) and (75) are Hamilton’s equations, and both are particular realizations of (73). The reason why we have passed from (47) to (75) is that the latter equation is more suitable for starting the reduction process. We assume we know from some other considerations (for instance, from experimental observations) that the pattern that represents the lower level in the phase portrait of (47) can be expressed in terms of Z. If this is the case, then clearly, the reformulation (75) of (47) is more suitable for investigating the reduction. Both (75) and (47) share the same kinematics, but the energies in them remain so far completely unrelated and at this point undetermined. Their determination is a part of the continuation of the pattern recognition process in the phase portrait of (47) that has to enter into an actual analysis of the solutions to (47).

In this paper, we make only a few comments about the physical aspects of the hierarchy (75). Let Z be the state variables used at the lower level. The hierarchy (75) thus governs the time evolution at the lower level. However, the time-evolution governed by (75) is still coupled to the time-evolution of *f*. We can physically interpret *f* as a state variable characterizing the overall features of the phase portrait of (47) that are not expressed in the lower state variables Z. An example of the considerations, based on physical assumptions and approximations, that lead to expressing *f* in terms of Z) is presented in the next section.

Still another insight into the physics expressed in the hierarchy (75) is revealed in its following reformulation. We note that the last equation in (75) (i.e., the equation governing the time-evolution of *f*) can be seen as the Liouville (i.e., continuity) equation corresponding to 6N ordinary differential equations governing the time-evolution of (1,…,N). We can thus reformulate (75) into a system (n+6N) of ordinary differential equations:

(76) $$\begin{array}{ccc} {\dot{Z}}_{\alpha } & = & \int d1\ldots \int dNf\left[\left(\frac{\partial z_{\alpha }}{\partial r_{\gamma i}}\frac{\partial z_{\beta }}{\partial v_{\gamma i}}-\frac{\partial z_{\beta }}{\partial r_{\gamma i}}\frac{\partial z_{\alpha }}{\partial v_{\gamma i}}\right)E_{Z_{\beta }}\right.\\ & & \left.+\left(\frac{\partial z_{\alpha }}{\partial r_{\gamma i}}\frac{\partial E_{f}}{\partial v_{\gamma i}}-\frac{\partial z_{\alpha }}{\partial v_{\gamma i}}\frac{\partial E_{f}}{\partial r_{\gamma i}}\right)\right]\\ {\dot{r}}_{\gamma i} & = & \frac{\partial z_{\alpha }}{\partial v_{\gamma i}}E_{Z_{\alpha }}+\frac{\partial E_{f}}{\partial v_{\gamma i}}\\ {\dot{v}}_{\gamma i} & = & -\frac{\partial z_{\alpha }}{\partial r_{\gamma i}}E_{Z_{\alpha }}-\frac{\partial E_{f}}{\partial r_{\gamma i}} \end{array}$$

that is accompanied by:

(77) $$f\left(1,\ldots ,N,t\right)=f_{0}\left(T_{-t}\left(1,\ldots ,N\right)\right)$$

where $T_{t}$ is the trajectory of (1,…,N) and $f_{0}\left(1,\ldots ,N\right)$ is an initial distribution function. In this formulation, we see clearly the role of *f*. It is indeed a state variable expressing the overall features of the dynamics that are expressed neither in (1,…,N), nor in Z.

Finally, we emphasize that all the reformulations that we have made above in this section do not involve any approximation. Both (47) and (75) share the same kinematics (but in different representations), and the energies in both equations remain undetermined. We can see (75) as a combination of the microscopic level represented by (47) and the mesoscopic level at which Z serve as state variables. In the next section, we take initial steps in the pattern recognition process leading to the closure of (75) (i.e., to expressing *f* in terms of Z).

### 5.1. Dissipation, Closure

In order to recognize a pattern in the phase portrait corresponding to (47) or to its reformulation (75), the phase portrait has to be first created (i.e., the solutions to (47) or to (75) have to be found). This can be done only if we specify the energy $E^{↑}\left(x\right)$ entering (47) or (75) and thus commit ourselves to specific macroscopic systems. An example of such an analysis was given in [10] for the Boltzmann equation. We shall not follow this path. Instead, we limit ourselves to some observations of a qualitative nature that combine physical and mathematical arguments.

First, we emphasize that the choice (72) of the lower state variables Z is already a part of the pattern recognition process. We anticipate that the pattern that we search in the phase portrait corresponding to (47) can be expressed in terms of Z. For example, we may recall the considerations leading to the choice of the hydrodynamic fields. Since the total mass, momentum, and energy are conserved, the local mass, momentum, and energy change at a slower pace than other mesoscopic state variables.

Having chosen (72), we follow the previous section and arrive at the hierarchy (75) (or (76)) combining the upper and lower levels. Now, we make a very obvious, but important observation. We can look at the hierarchy in two ways: “bottom up” and “top down”. In (75), the bottom part is the first equation, and the top part is the second equation. In infinite hierarchies, the second equation is replaced by an infinite hierarchy of equations governing the time-evolution of $\left(Z_{n+1},Z_{n+2},\ldots \right)$.

The standard view is bottom up. It is the reduced dynamics, which is an appropriately closed bottom part of the hierarchy, which is typically the reason why the reductions are made. We look at the bottom part of the hierarchy and try to close it. In the hierarchy (75), this means that we look at the first equation and try to express *f* appearing in it in terms of Z (or in infinite hierarchies in terms of $\left(Z_{n+1},Z_{n+2},\ldots )\right)$. The closure can be argued by putting various requirements on the closed system of equations. In our analysis, the natural requirement is that the closed system of equations remains to be a system of Hamilton’s equations. In the case of dissipative dynamics, we may require that an appropriate entropy (a real-valued function of Z) will not decrease during the time-evolution governed by the closed system of equations. This latter requirement first appeared in [82] and was later developed in [96].

However, the closure can also be argued from top down. As we already noticed in Section 2.5, this viewpoint of the closure is in fact present in the Chapman–Enskog method, and it was also compared with the bottom up viewpoint in [24] (where, however, only the static MaxEnt version of the top part of the hierarchy was considered). In this paper, we continue to explore the top down view of the closure.

The top part of the hierarchy (75) is its second equation in which $z_{1}\left(1,\ldots ,N\right),\ldots ,z_{n}\left(1,\ldots ,N\right)$ are seen as quantities representing external influences in the time-evolution of *f*. If (75) is cast into the form (76), then the top part consists of the last two equations in (76) together with (77). The quantities Z appear in (76) indeed as extra velocities and extra forces in the Hamilton time-evolution of (1,…,N). In order to close the hierarchy (75), we have to find the phase portrait corresponding to its top part and then recognize in it a pattern parametrized by Z. In other words, we have to express *f* in terms of Z (we denote it by $\hat{f}\left(Z;1,\ldots ,N\right)$) by analyzing the solutions of the top part of the hierarchy. By inserting $\hat{f}\left(Z;1,\ldots ,N\right)$ into the lower part of the hierarchy (i.e., into the first equation in (75)), we arrive at the lower dynamics.

Such investigation cannot be done without making a commitment to a specific physical system and without a type of analysis displayed for example in [10]. In the rest of this section, we make only a very qualitative analysis that transforms the bottom part of (75) with the Onsager time-evolution equation.

We begin by noting that S(f)=∫d1…∫dNη(f), where η:R→R is a sufficiently regular function, is a Casimir of the Poisson bracket (74). We can therefore put (76) into the form:

(78) $$\begin{array}{ccc} {\dot{Z}}_{\alpha } & = & \int d1\ldots \int dNf\left[\left(\frac{\partial z_{\alpha }}{\partial r_{\gamma i}}\frac{\partial z_{\beta }}{\partial v_{\gamma i}}-\frac{\partial z_{\beta }}{\partial r_{\gamma i}}\frac{\partial z_{\alpha }}{\partial v_{\gamma i}}\right)\Phi_{Z_{\beta }}\right.\\ & & \left.+\left(\frac{\partial z_{\alpha }}{\partial r_{\gamma i}}\frac{\partial \Phi_{f}}{\partial v_{\gamma i}}-\frac{\partial z_{\alpha }}{\partial v_{\gamma i}}\frac{\partial \Phi_{f}}{\partial r_{\gamma i}}\right)\right]\\ \frac{\partial f}{\partial t} & = & -\frac{\partial }{\partial r_{\gamma i}}\left(f\frac{\partial z_{\alpha }}{\partial v_{\gamma i}}\right)\Phi_{Z_{\alpha }}+\frac{\partial }{\partial v_{\gamma i}}\left(f\frac{\partial z_{\alpha }}{\partial r_{\gamma i}}\right)\Phi_{Z_{\alpha }}\\ & & -\frac{\partial }{\partial r_{\gamma i}}\left(f\frac{\partial \Phi_{f}}{\partial v_{\gamma i}}\right)+\frac{\partial }{\partial v_{\gamma i}}\left(f\frac{\partial \Phi_{f}}{\partial r_{\gamma i}}\right) \end{array}$$

where $\Phi \left(Z,f\right)=-S\left(f\right)+\frac{1}{T}E\left(Z,f\right)$ and the constant temperature *T* is absorbed in rescaling the time. In the next step, we introduce to the top equation in the hierarchy (i.e., to the second equation in (78)) a dissipation. From physical considerations, we anticipate that the dominant dissipation is the Fokker–Planck-type diffusion in momenta. If we restrict ourselves to the linear dissipation (i.e., if we restrict ourselves to states that are not far from equilibrium states), the top part of the hierarchy (78) becomes:

(79) $$\begin{array}{ccc} \frac{\partial f}{\partial t} & = & -\frac{\partial }{\partial r_{\gamma i}}\left(f\frac{\partial z_{\alpha }}{\partial v_{\gamma i}}\right)\Phi_{Z_{\alpha }}+\frac{\partial }{\partial v_{\gamma i}}\left(f\frac{\partial z_{\alpha }}{\partial r_{\gamma i}}\right)\Phi_{Z_{\alpha }}\\ & & -\frac{\partial }{\partial r_{\gamma i}}\left(f\frac{\partial \Phi_{f}}{\partial v_{\gamma i}}\right)+\frac{\partial }{\partial v_{\gamma i}}\left(f\frac{\partial \Phi_{f}}{\partial r_{\gamma i}}\right)\\ & & -\frac{\partial }{\partial v_{\gamma i}}\left(f\lambda_{\gamma \delta ij}\frac{\partial \Phi_{f}}{\partial v_{\delta j}}\right) \end{array}$$

where λ is a symmetric matrix guaranteeing $\int d1\ldots \int dNf\frac{\partial \Phi_{f}}{\partial v_{\gamma i}}\lambda_{\gamma \delta ij}\frac{\partial \Phi_{f}}{\partial v_{\delta j}}\geq 0$.

To continue, we use physical considerations to identify dominant terms on the right-hand side of (79). We recall that this type of argument is also the point of departure of the Chapman–Enskog passage from the Boltzmann kinetic equations to fluid mechanics (see Section 4.1). The anticipated dominance of variations in momenta (which can be physically interpreted as an anticipation of the occurrence of turbulence at the micro scale), which led us already to the introduction of the dissipative term in (79), leads us to regard the second term on the right-hand side of (79) as dominant. We thus assume that the main part of (79) is the time-evolution equation:

(80) $$\frac{\partial f}{\partial t}=\frac{\partial }{\partial v_{\gamma i}}\left(f\frac{\partial z_{\alpha }}{\partial r_{\gamma i}}\right)\Phi_{Z_{\alpha }}-\frac{\partial }{\partial v_{\gamma i}}\left(f\lambda_{\gamma \delta ij}\frac{\partial \Phi_{f}}{\partial v_{\delta j}}\right)$$

We have omitted the term $\frac{\partial }{\partial r_{\gamma i}}\left(f\frac{\partial \Phi_{f}}{\partial v_{\gamma i}}\right)$, which we assume to be smaller than the remaining two terms. Next, we assume that the distribution function *f* evolves faster than Z and that we are already in the stage of the time-evolution in which *f* reaches the stationary state.

We need now to solve:

(81) $$0=\frac{\partial }{\partial v_{\gamma i}}\left(f\frac{\partial z_{\alpha }}{\partial r_{\gamma i}}\right)\Phi_{Z_{\alpha }}-\frac{\partial }{\partial v_{\gamma i}}\left(f\lambda_{\gamma \delta ij}\frac{\partial \Phi_{f}}{\partial v_{\delta j}}\right)$$

In order to avoid complications with the degeneracy of the matrix λ (which is needed to satisfy the energy conservation), we limit ourselves in this paper to isothermal systems. The thermodynamic potential Φ is thus the Helmholtz free energy, and the matrix λ is a positive definite matrix, which can be inverted. We can thus easily solve (81). If we insert the solution into the first equation in (78) (in which we omit the term $\frac{\partial }{\partial r_{\gamma i}}\left(f\frac{\partial z_{\alpha }}{\partial v_{\gamma i}}\right)$ that we assume to be small relative to the other terms in (78)), we obtain:

(82) $${\dot{Z}}_{\alpha }=L_{\alpha \beta }\Phi_{Z_{\beta }}-\Lambda_{\alpha \beta }\Phi_{Z_{\beta }}$$

where:

(83) $$\begin{array}{ccc} L_{\alpha \beta } & = & \int d1\ldots \int dNf\left(\frac{\partial z_{\alpha }}{\partial r_{\gamma i}}\frac{\partial z_{\beta }}{\partial v_{\gamma i}}-\frac{\partial z_{\beta }}{\partial r_{\gamma i}}\frac{\partial z_{\alpha }}{\partial v_{\gamma i}}\right) \end{array}$$

(84) $$\begin{array}{ccc} \Lambda_{\alpha \beta } & = & \int d1\ldots \int dNf\frac{\partial z_{\alpha }}{\partial r_{\gamma i}}\lambda_{\gamma \delta ,ij}^{-1}\frac{\partial z_{\beta }}{\partial r_{\delta j}} \end{array}$$

Equation (82) governing the time-evolution of Z still needs to be closed. The distribution functions *f* still appears in the matrices *L* and Λ in (83) and (84). We assume now that we have independent information about the overall state of the system under investigation and thus about *f*. For instance, if the system under investigation is close to equilibrium, we can replace *f* in (83) and (84) by the Gibbs equilibrium distribution function.

Considering *f* in (83) and (84) to be known, Equation (82) is a closed equation governing the time-evolution of the mesoscopic state variables Z. Equation (82) is the Onsager equation [81]. The matrix Λ is symmetric, positive definite, and does not change its sign if the momenta $\left(v_{1},\ldots ,v_{N}\right)$ change their signs. The matrix *L* is skew-symmetric, and it changes its sign if $\left(v_{1},\ldots ,v_{N}\right)\to \left(-v_{1},\ldots ,-v_{N}\right)$. Equation (82) is also a GENERIC equation since (82) is a particular realization of (12). The matrix *L* is indeed skew-symmetric, and the Poisson bracket {A,B}= $A_{Z_{\alpha }}L_{\alpha \beta }B_{Z_{\beta }}$ satisfies the Jacobi identity since *L* is independent of Z. We note that the only unspecified parameter in the Formulas (83) and (84) for the matrices *L* and Λ is the matrix λ (entering the microscopic time-evolution (81)).

Summing up, the Onsager [81] result (82) has emerged as a part of the reduced structure at the lower level with the state variables Z. The reduction is made by reducing the time-evolution that preserves the Hamiltonian structure of the time-evolution that takes place at the upper level. We note in particular that the Onsager symmetry of Λ is a direct consequence of the gradient structure at the upper level that guarantees the existence of the reduction. The skew-symmetry of *L* and its sign change in the transformation $\left(v_{1},\ldots ,v_{N}\right)\to \left(-v_{1},\ldots ,-v_{N}\right)$ are a direct consequence of the Hamiltonian structure of the upper level time-evolution. In the absence of dissipation, Equation (82) represents GENERIC dynamics.

We can also find the lower rate thermodynamic relation $\Sigma^{↓}\left(y\right)$ implied by the reduction discussed above. We note that with:

(85) $$\Sigma^{↑}\left(X\right)=-\frac{1}{2}\int d1\ldots \int dNfX_{\gamma i}\lambda_{\gamma \delta ij}X_{\delta j}$$

we can write (81) as:

(86) $$\Psi_{X}^{↑}\left(X,Y\right)=0$$

where:

(87) $$\Psi^{↑}\left(X,Y\right)=-\Sigma^{↑}\left(X\right)+\int d1\ldots \int dNfY_{\gamma i}X_{\gamma i}$$

with:

(88) $$Y_{\gamma i}=\frac{\partial z_{\alpha }}{\partial r_{\gamma i}}\Phi_{Z_{\alpha }}$$

Consequently, the lower rate entropy implied by the above reduction is:

(89) $$\Sigma^{↓}\left(Y\right)=\frac{1}{2}\int d1\ldots \int dNf\frac{\partial z_{\alpha }}{\partial r_{\gamma i}}\lambda_{\gamma \delta ,ij}^{-1}\frac{\partial z_{\beta }}{\partial r_{\delta j}}$$

We note that Equation (82) implies:

(90) $$\dot{\Phi }=\Phi_{Z_{\alpha }}{\dot{Z}}_{\alpha }=\frac{1}{2}\Sigma^{↓}$$

which relates the lower rate entropy to the lower entropy production.

### 5.2. Illustrations

The Liouville Equation (47) governing the Hamiltonian time-evolution of the N particle distribution function, $N∼10^{23}$, was the first equation that was cast into the hierarchy form. The hierarchy reformulation of the Liouville Equation (47) is called the BBGKYhierarchy [93,97]. Another time-evolution equation that gave rise to a famous hierarchy (Grad hierarchy [48]) is the Boltzmann kinetic equation. Below, we shall cast into the hierarchy also the Euler hydrodynamic equation. The kinematics-preserving hierarchies for all three equations illustrate the general analysis presented above. In all three hierarchies, we limit ourselves in this paper only to the Hamiltonian part of the time-evolution.

#### 5.2.1. BBGKY Kinematics-Preserving Hierarchy

The state variable at the upper level is the N particle distribution function $f_{N}\left(1,\ldots ,N\right)$. The lower level state variables are 1,…,N−1 distribution functions obtained from $f_{N}$ by integrating over the N−1,…,2 coordinates, respectively. All distribution functions are symmetric with respect to relabeling the particles. The kinematics of $f_{N}$ is expressed in the Poisson bracket (46).

The infinite kinematics-preserving hierarchy in this setting was developed in [98] for 1,…,N,… distribution functions. Here, we present a kinematics-preserving hierarchy reformulation of the Liouville equation governing the time-evolution of $f_{2}\left(1,2\right)$. To simplify the notation, we use *f* to denote $f_{2}$ and *g* to denote $f_{1}$. The one particle distribution function *g* is related to *f* by:

(91) $$g\left(r,v\right)=\int d1\int d2f\left(1,2\right)\left[\delta \left(r-r_{1}\right)\delta \left(v-v_{1}\right)+\delta \left(r-r_{2}\right)\delta \left(v-v_{2}\right)\right]$$

In order to find the kinematics of (f,g), we take the Poisson bracket (46) for N=2 with the functions *A* and *B* that depend on *f* directly and also indirectly through their dependence on *g* (which depends on *f*; see (91)). By replacing $A_{f}$ in (46) with $A_{f(1,2)}=A_{g(1)}+A_{g(2)}+A_{f(1,2)}$ and similarly $B_{f(1,2)}$, the Poisson bracket (46) becomes the Poisson bracket:

(92) $$\begin{array}{ccc} \left\{A,B\right\} & = & {\left\{A,B\right\}}^{\left(N=1\right)}\\ & & +\int d1\int d2\left[f\left(\frac{\partial A_{f}}{\partial r_{1i}}\frac{\partial B_{g(1)}}{\partial v_{1i}}-\frac{\partial B_{f}}{\partial r_{1i}}\frac{\partial A_{g(1)}}{\partial v_{1i}}\right)\right.\\ & & +\left.f\left(\frac{\partial A_{f}}{\partial r_{2i}}\frac{\partial B_{g(2)}}{\partial v_{2i}}-\frac{\partial B_{f}}{\partial r_{2i}}\frac{\partial A_{g(2)}}{\partial v_{2i}}\right)\right.\\ & & +\left.f\left(\frac{\partial A_{g(1)}}{\partial r_{1i}}\frac{\partial B_{f}}{\partial v_{1i}}-\frac{\partial B_{g(1)}}{\partial r_{1i}}\frac{\partial A_{f}}{\partial v_{1i}}\right)\right.\\ & & +\left.f\left(\frac{\partial A_{g(2)}}{\partial r_{2i}}\frac{\partial B_{f}}{\partial v_{2i}}-\frac{\partial B_{g(2)}}{\partial r_{2i}}\frac{\partial A_{f}}{\partial v_{2i}}\right)\right]\\ & & +{\left\{A,B\right\}}^{\left(N=2\right)} \end{array}$$

where ${\left\{A,B\right\}}^{\left(N=1\right)}$ and ${\left\{A,B\right\}}^{\left(N=2\right)}$ are the Poisson brackets (46) for N=1 and N=2, respectively. There is an important difference between the Poisson bracket (92) and the bracket (74). In (92), the bracket is the sum of three brackets, one involving only the lower state variable *g*, the other involving both the upper *f* and the lower *g* state variables, and the third involves only the upper state variable *f*. The bracket (74) is the sum of two terms, one involving both the upper and the lower state variables and the other only the upper state variable.

The time-evolution Equation (73) corresponding to the Poisson bracket (92) becomes:

(93) $$\begin{array}{ccc} \frac{\partial g}{\partial t} & = & -\frac{\partial }{\partial r_{i}}\left(g\frac{\partial E_{g}}{\partial v_{i}}\right)+\frac{\partial }{\partial v_{i}}\left(g\frac{\partial E_{g}}{\partial r_{i}}\right)\\ & & +2\int dr_{2}\int dv_{2}\left[-\frac{\partial }{\partial r_{i}}\left(f\frac{\partial E_{f}}{\partial v_{i}}\right)+\frac{\partial }{\partial v_{i}}\left(f\frac{\partial E_{f}}{\partial r_{i}}\right)\right]\\ \frac{\partial f}{\partial t} & = & \sum_{\alpha =1}^{2}\left[\frac{\partial }{\partial r_{\alpha i}}\left(f\frac{\partial E_{g}}{\partial v_{\alpha i}}\right)+\frac{\partial }{\partial v_{\alpha i}}\left(f\frac{\partial E_{g}}{\partial r_{\alpha i}}\right)\right]\\ & & +\sum_{\alpha =1}^{2}\left[-\frac{\partial }{\partial r_{\alpha i}}\left(f\frac{\partial E_{f}}{\partial v_{\alpha i}}\right)+\frac{\partial }{\partial v_{\alpha i}}\left(f\frac{\partial E_{f}}{\partial r_{\alpha i}}\right)\right] \end{array}$$

The energy E(f,g) remains in these equations undetermined.

Now, we discuss qualitative properties of solutions to (93). We note that if the energy E(f,g) is chosen to be independent of *f*, then (93) turns into the standard one particle Hamiltonian kinetic equation. This is a consequence of the presence of the bracket ${\left\{A,B\right\}}^{\left(N=1\right)}$ (that does not involve *f*) on the right-hand side of (92). If, on the other hand, the energy E(f,g) is independent of *g*, then (93) becomes the two particle Hamiltonian kinetic equation. For the energy *E* that depends on both *g* and *f*, the time-evolution of the one and the two particle distribution functions are coupled, and the two particle distribution function *f* represents extra details that are ignored in the one particle kinetic theory.

We also note that the kinematics-preserving hierarchy (93) is different from the classical BBGKY hierarchy:

(94) $$\begin{array}{ccc} \frac{\partial g(r,v)}{\partial t} & = & -\frac{\partial }{\partial r_{i}}\left(2\int d2f\left(r,v,r_{2},v_{2}\right)\frac{\partial E_{f(r,v,r_{2},v_{2})}}{\partial v_{i}}\right)\\ & & +\frac{\partial }{\partial v_{i}}\left(2\int d2f\left(r,v,r_{2},v_{2}\right)\frac{\partial E_{f(r,v,r_{2},v_{2})}}{\partial r_{i}}\right)\\ \frac{\partial f(1,2)}{\partial t} & = & \sum_{\alpha =1}^{2}\left[-\frac{\partial }{\partial r_{\alpha i}}\left(f\frac{\partial E_{f}}{\partial v_{\alpha i}}\right)+\frac{\partial }{\partial v_{\alpha i}}\left(f\frac{\partial E_{f}}{\partial r_{\alpha i}}\right)\right] \end{array}$$

where *E* is a function of *f*. The classical BBGKY hierarchy is obtained from Equation (47) with N=2 by simply integrating it over ∫d2. We recall that the point of departure for obtaining the kinematics-preserving hierarchy (93) is the kinematics (46) with N=2, while the point of departure for the classical hierarchy (94) is the two particle Liouville Equation (47). The original Equation (47) with N=2 is Hamiltonian; its classical hierarchy reformulation (94) is not Hamiltonian, but its kinematics-preserving hierarchy (93) keeps the Hamiltonian structure.

#### 5.2.2. Grad Kinematics-Preserving Hierarchy

For the second illustration, we turn to the Boltzmann equation governing the time-evolution of the one particle distribution function f(r,v) and to the time-evolution of its Grad moments:

(95) $$f\left(r,v\right)\mapsto (c^{\left(0\right)}\left(r\right),c_{i}^{\left(1\right)}\left(r\right),\ldots ,c_{i_{1},\ldots ,i_{k}}^{\left(k\right)}\left(r\right),\ldots )$$

where:

(96) $$c_{i_{1},\ldots ,i_{k}}^{\left(k\right)}\left(r\right)=\int dvv_{i_{1}}\ldots v_{i_{k}}f\left(r,v\right)$$

The kinematics of *f* is expressed in the Poisson bracket (46) with N=1.

The infinite kinematics-preserving hierarchy with an infinite number of Grad moments (96) as lower state variables was worked out in [99]. Here, we present a Grad kinematics-preserving five moment hierarchy closed by an equation governing the time-evolution of *f*.

We choose (95) and (96) with:

(97) $$\begin{array}{ccc} & & f(r,v)\mapsto (\rho (r),u(r),s(r))\\ & & \rho (r)=\int dvf(r,v);\;\;u(r)=\int dvvf(r,v);\;\;s(r)=\int dv\eta (f(r,v)) \end{array}$$

where η(f) is a sufficiently regular function R→R. We recall that (see Section 4.1) ∫dr∫dvη(f) is a Casimir (see Section 2.1.1) of the Poisson bracket (46) with N=1. We choose the hydrodynamic state variables in the energy representation (i.e., the state variables are the fields (ρ(r),u(r),s(r)) denoting the mass, momentum, and entropy) rather than in the entropy representation with the state variables (ρ(r),u(r),e(r), where e(r) is the energy field. The reason for the choice is explained in Section 4.3.2.

From the kinematics of the kinetic theory that is expressed in the Poisson bracket (46) with N=1, we derive the kinematics-preserving hierarchy in the same way as in the previous two sections. The functions *A* and *B* in (46) with N=1 depend on *f* directly and also through their dependence on the moments (97). Consequently, we replace $A_{f}$ and $B_{f}$ with $A_{\rho (r)}+v_{i}A_{u_{i}\left(r\right)}+\eta_{f(r,v)}A_{s(r)}+A_{f(r,v)}$ and the same expression for $B_{f}$. Straightforward calculations then lead to the Poisson bracket:

(98) $$\begin{array}{ccc} {\left\{A,B\right\}}^{\left(kh\right)} & = & {\left\{A,B\right\}}^{\left(hyd\right)}\\ & & +\int dr\int dv\left[f\left(\frac{\partial A_{f}}{\partial r_{i}}B_{u_{i}}-\frac{\partial B_{f}}{\partial r_{i}}A_{u_{i}}\right)\right.\\ & & \left.+f\frac{\partial \eta_{f}}{\partial v_{i}}\left(\frac{\partial A_{f}}{\partial r_{i}}B_{s}-\frac{\partial B_{f}}{\partial r_{i}}A_{s}\right)\right.\\ & & \left.+f\left(\frac{\partial A_{\rho }}{\partial r_{i}}\frac{\partial B_{f}}{\partial v_{i}}-\frac{\partial B_{\rho }}{\partial r_{i}}\frac{\partial A_{f}}{\partial v_{i}}\right)\right.\\ & & \left.+fv_{j}\left(\frac{\partial A_{u_{j}}}{\partial r_{i}}\frac{\partial B_{f}}{\partial v_{i}}-\frac{\partial B_{u_{j}}}{\partial r_{i}}\frac{\partial A_{f}}{\partial v_{i}}\right)\right.\\ & & \left.+f\left(\frac{\partial (A_{s}\eta_{f})}{\partial r_{i}}\frac{\partial B_{f}}{\partial v_{i}}-\frac{\partial (B_{s}\eta_{f})}{\partial r_{i}}\frac{\partial A_{f}}{\partial v_{i}}\right)\right]\\ & & +{\left\{A,B\right\}}^{\left(N=1\right)} \end{array}$$

where ${\left\{A,B\right\}}^{\left(hyd\right)}$ is the Poisson bracket (57) expressing the kinematics of fluids and ${\left\{A,B\right\}}^{\left(N=1\right)}$ is the Poisson bracket (46) with N=1. Like the Poisson bracket (92), but unlike the Poisson bracket (74), the Poisson bracket (98) is the sum of three brackets, one depending only on the lower, the other only on the upper, and only the third on both the lower and the upper state variables. As we have already discussed in Section 4.3.2, the hydrodynamic bracket ${\left\{A,B\right\}}^{\left(hyd\right)}$ appears in the kinematics-preserving hierarchy bracket only with the hydrodynamic moments (97). With any other choice of the moments (e.g., if the entropy field s(r) is replaced by the energy field e(r)=∫dvϵ(r,v)f(r,v), where ϵ(r,v) is a microscopic energy), the one particle distribution function *f* will still remain in all terms in the Poisson bracket.

The time-evolution equations (3) with the Poisson bracket (98) and energy:

(99) E(f,ρ,u,s)=∫dre(f,ρ,u,s;r)=∫dr∫dvϵ(f,ρ,u,s;r,v)

are:

(100) $$\begin{array}{ccc} \frac{\partial \rho }{\partial t} & = & -\frac{\partial }{\partial r_{i}}\left(\rho E_{u_{i}}+\int dvf\frac{\partial E_{f}}{\partial v_{i}}\right)\\ \frac{\partial s}{\partial t} & = & -\frac{\partial }{\partial r_{i}}\left(sE_{u_{i}}+\int dv\eta \frac{\partial E_{f}}{\partial v_{i}}\right)\\ \frac{\partial u_{i}}{\partial t} & = & -\frac{\partial }{\partial r_{j}}\left(u_{i}E_{u_{j}}+\int dvfv_{i}\frac{\partial E_{f}}{\partial v_{j}}\right)\\ & & +\frac{\partial }{\partial r_{i}}\left(-\int dv\epsilon +\rho E_{\rho }+sE_{s}+u_{j}E_{u_{j}}+\int dvfE_{f}\right)\\ \frac{\partial f}{\partial t} & = & -\frac{\partial }{\partial r_{i}}\left(fE_{u_{i}}+f\frac{\partial \eta_{f}}{\partial v_{i}}E_{s}\right)\\ & & +\frac{\partial }{\partial v_{i}}\left(f\frac{\partial E_{\rho }}{\partial r_{i}}+f\frac{\partial (E_{s}\eta_{f})}{\partial r_{i}}+fv_{j}\frac{\partial E_{u_{i}}}{\partial r_{j}}\right)\\ & & -\frac{\partial }{\partial r_{i}}\left(f\frac{\partial E_{f}}{\partial v_{i}}\right)+\frac{\partial }{\partial v_{i}}\left(f\frac{\partial E_{f}}{\partial r_{i}}\right) \end{array}$$

This kinematics-preserving hierarchy was already derived in [100].

The disadvantage of the choice of the energy representation (i.e., the disadvantage of the choice of the state variables (ρ(r),s(r),u(r))) is that the transformation to the entropy representation with the state variables (ρ(r),u(r),e(r)), which is more suitable in most applications, requires an additional assumption. The passage (ρ(r),u(r),s(r))→(ρ(r),u(r),e(ρ(r),u(r),s(r)) is one-to-one only if $\frac{\partial e(r)}{\partial s(r)}>0$. The assumption that $\frac{\partial e(r)}{\partial s(r)}>0$ is in fact a weak form of the local equilibrium assumption. According to this assumption, the quantities s(r) and e(r) are the (local) equilibrium entropy and (local) energy, respectively, and consequently, $\frac{\partial e(r)}{\partial s(r)}=T\left(r\right)>0$ is the (local) absolute (and thus positive) temperature. We recall that when passing from one representation to another, the gradients change as follows: $E_{s}=\frac{1}{S_{e}};E_{u}=-\frac{S_{u}}{S_{e}}$.

If there is a one-to-one relation between the energy and the entropy representations, then the time-evolution of the energy field e(r) is governed by:

(101) $$\begin{array}{ccc} \frac{\partial e}{\partial t} & = & -\frac{\partial }{\partial r_{i}}\left[\rho E_{\rho }E_{u_{i}}+sE_{s}E_{u_{i}}+u_{j}E_{u_{j}}E_{u_{i}}\right.\\ & & \left.+\int dv\left(fE_{f}E_{u_{i}}+fE_{\rho }\frac{\partial E_{f}}{\partial v_{i}}+\eta E_{s}\frac{\partial E_{f}}{\partial v_{i}}+fE_{f}\frac{\partial E_{f}}{\partial v_{i}}\right)\right] \end{array}$$

We turn now to the qualitative properties of the solutions of (100). A direct consequence of the presence of the term ${\left\{A,B\right\}}^{\left(hyd\right)}$ on the right-hand side of the Poisson bracket (98) (a term that does not involve *f*) is that the hierarchy (100) reduces to the standard non-dissipative hydrodynamic equations if the energy *E* is chosen to be independent of *f*, On the other hand, if *E* is chosen to depend only on *f*, then (100) becomes the non-dissipative one particle kinetic equation. For a general energy *E* depending on both the hydrodynamic fields and the one particle distribution function *f*, the kinematics-preserving hierarchy (100) represents a Hamiltonian extended hydrodynamics in which the one particle distribution function plays the role of an extra state variable *f*. The specific physical interpretation of *f* is determined by the specification of the energy E(f,g), i.e., by the role that *f* plays in the forces driving the time-evolution.

Having the hierarchy reformulation (100) of the kinetic equation (or other hierarchy reformulations discussed in the previous two sections), how can we use it to make the MaxRent passage to the hydrodynamic equations? A detailed analysis of the solutions of (100), in particular an analysis of the onset of irregularities in solutions (see e.g., [10]), is expected to lead us to the upper rate entropy $\Sigma^{↑}$ generating the dissipation that eventually, by following the dissipative time-evolution, eliminates the details expressed in *f* and leaves us only with equations governing the time-evolution of hydrodynamic fields. An example of this type of physical consideration, but in the context of MaxEnt not MaxRent, is Boltzmann’s realization that the binary collisions are responsible for the onset of the irregularities of solutions of the Hamilton one particle kinetic equation and for the emergence of dissipation in its regularized solutions. We hope to follow this route in the future.

In this paper, we only note that already, the hierarchy reformulation (100) is useful in determining the upper rate fundamental thermodynamic relation. The vector field $J^{↑}\left(f;\rho ,s,u\right)$ is read in the second terms on the right-hand side of the equations governing the time-evolution of the hydrodynamic fields. Moreover, the first two lines in the equations governing the time evolution of *f* in (100) indicate also $J^{*}\left(\rho ,s,u\right)$. However, we emphasize that the passage from (100) to the upper rate fundamental thermodynamic relation and to a proof that solutions to (100) modified by supplying it with the dissipative term approach the upper equilibrium state $\overset{˘}{x}$ is left unsolved in this paper.

#### 5.2.3. Euler Kinematics-Preserving Hierarchy

All three examples (75), (93), and (100) of kinematics-preserving hierarchies are hierarchy reformulations of equations governing the time-evolution of distribution functions. In this last illustration, we present the kinematics-preserving hierarchy of the Euler hydrodynamic equation. The upper level is the level of fluid mechanics; the upper state variable is the momentum field u(r); and the Poisson bracket:

(102) $$\left\{A,B\right\}=\int dru_{i}\left(\frac{\partial A_{u_{i}}}{\partial r_{j}}B_{u_{j}}-\frac{\partial B_{u_{i}}}{\partial r_{j}}A_{u_{j}}\right)$$

expresses mathematically its kinematics. The lower level state variables

$W=(W_{1},\ldots ,W_{n})$ are introduced by:

(103) $$W_{\alpha i}=\int drw_{\alpha }\left(r\right)u_{i}\left(r\right)$$

where $\left(w_{1}\left(r\right),\ldots ,w_{n}\left(r\right)\right)$ is a given fixed set of *n* functions $R^{3}\to R$.

In order to reformulate the Euler equation as a kinematics-preserving hierarchy involving W (see (103)) as the lower level state variables, we proceed as in the previous sections. The functions *A* and *B* in (102) depend on *u* directly and also indirectly through the dependence on W (which depends on u(r); see (103)). We thus replace $A_{u}$ and $B_{u}$ in (102) with $A_{u_{i}\left(r\right)}=w_{\alpha }\left(r\right)A_{W_{\alpha i}}+A_{u_{i}\left(r\right)}$ and with the same expression for $B_{u}$. This change transforms (102) into:

(104) $$\begin{array}{ccc} \left\{A,B\right\} & = & \int dr\left[u_{i}w_{\beta }\frac{\partial w_{\alpha }}{\partial r_{j}}\left(A_{W_{\alpha i}}B_{W_{\beta j}}-B_{W_{\alpha i}}A_{W_{\beta j}}\right)\right.\\ & & \left.+u_{i}\frac{\partial w_{\alpha }}{\partial r_{j}}\left(A_{W_{\alpha i}}B_{u_{j}}-B_{W_{\alpha i}}A_{u_{j}}\right)\right.\\ & & \left.+u_{i}w_{\beta }\left(\frac{\partial A_{u_{i}}}{\partial r_{j}}B_{W_{\beta j}}-\frac{\partial B_{u_{i}}}{\partial r_{j}}A_{W_{\beta j}}\right)\right.\\ & & \left.+u_{i}\left(\frac{\partial A_{u_{i}}}{\partial r_{j}}B_{u_{j}}-\frac{\partial B_{u_{i}}}{\partial r_{j}}A_{u_{j}}\right)\right] \end{array}$$

In this Poisson bracket (as well as in the Poisson bracket (74)), the upper state variable *u* appears in all its terms. The Poisson brackets (92) and (98) involving only the lower state variables are rather exceptional.

The time-evolution equations corresponding to this bracket is the following kinematics-preserving hierarchy:

(105) $$\begin{array}{ccc} {\dot{W}}_{\alpha i} & = & \int dr\left[\left(u_{i}w_{\beta }\frac{\partial w_{\alpha }}{\partial r_{j}}-u_{j}w_{\alpha }\frac{\partial w_{\beta }}{\partial r_{i}}\right)E_{W_{\beta j}}\right.\\ & & \left.-u_{j}w_{\alpha }\frac{\partial E_{u_{j}}}{\partial r_{i}}+u_{i}\frac{\partial w_{\alpha }}{\partial r_{j}}E_{u_{j}}\right]\\ \frac{\partial u_{i}}{\partial t} & = & -\frac{\partial }{\partial r_{j}}\left(u_{i}E_{u_{j}}+u_{i}w_{\beta }E_{W_{\beta j}}\right)-\frac{\partial p}{\partial r_{i}} \end{array}$$

where $p=-e+u_{j}w_{\beta }E_{W_{\beta j}}+u_{j}E_{u_{j}}$ and E(u,W)=∫dre(u,W;r). The momentum field u(r) appearing in (105) can be physically interpreted as an average momentum field and ***W*** as its fine internal structure. The possible suitability of this reformulation of the Euler equation for, for example, turbulence modeling or numerical investigations is intended to be explored in a future paper.

## 6. Concluding Remarks

Multiscale thermodynamics is a theory of relations among the levels of investigation of complex systems. It is a theory that sprung from the classical equilibrium thermodynamics, Boltzmann’s kinetic theory, and the Gibbs equilibrium statistical mechanics. A level is well established if its predictions agree with the results of experimental observations. A level L is an upper level vis-á-vis another level *l* if L includes more details than *l*. If both levels L and *l* are well established, then there must exist a way to prepare the systems under investigation for the level *l*, and the preparation process has to be possible to be seen as a time-evolution at the level L The entropy appearing in the vector field governing such a time-evolution plays at the level L the role of an ambassador of the lower level *l*. During the time-evolution, the entropy is maximized subject to certain constraints that, as well as the entropy, represent the lower level *l* inside the upper level L. The time-evolution in L leading to *l* is a sequence of infinitesimal contact structure-preserving transformations, and the whole process of passing from the level L to the level *l* is a reducing Legendre transformation. Multiscale thermodynamics investigates the chain ⟶L⟶L⟶l⟶, where L is a level that involves more details than both levels L and *l*. More generally, the chain is replaced by an oriented graph with levels as its vertices and links, directed toward lower levels, as reductions.

In this paper, we first present the main tenets of multiscale thermodynamics (in Section 2 and Section 3), and then, in Section 4, we show its realizations in the setting of classical theories like Boltzmann’s kinetic theory, Gibbs equilibrium statistical mechanics, and fluid mechanics. Dynamic and static theories at a wide range of scales become particular realizations of a single abstract theory applicable to externally and internally unforced and forced complex systems with no limitations regarding the closeness to equilibrium. In Section 5, we turn multiscale thermodynamics towards a new path in hierarchy reformulations of dynamical theories. Our objective is to formulate hierarchies that preserve kinematics. In both the classical and the newly explored theories, the multiscale thermodynamics inspires novel insights and viewpoints.
